# Anionic Lipid Nanoparticles Preferentially Deliver mRNA to the Hepatic Reticuloendothelial System

**DOI:** 10.1002/adma.202201095

**Published:** 2022-03-10

**Authors:** Roy Pattipeiluhu, Gabriela Arias-Alpizar, Genc Basha, Karen Y. T. Chan, Jeroen Bussmann, Thomas H. Sharp, Mohammad-Amin Moradi, Nico Sommerdijk, Edward N. Harris, Pieter R. Cullis, Alexander Kros, Dominik Witzigmann, Frederick Campbell

**Affiliations:** Supramolecular and Biomaterials Chemistry, Leiden Institute of Chemistry, Leiden University, Leiden 2333 CC, The Netherlands; BioNanoPatterning, Department of Cell and Chemical Biology, Leiden University Medical Center, Leiden 2333 RC, The Netherlands; Supramolecular and Biomaterials Chemistry, Leiden Institute of Chemistry, Leiden University, Leiden 2333 CC, The Netherlands; Division of BioTherapeutics, Leiden Academic Centre for Drug Research, Leiden University, Leiden 2333 CC, The Netherlands; NanoMedicines Research Group, Department of Biochemistry and Molecular Biology, University of British Columbia, Vancouver V6T 1Z3, Canada; NanoMedicines Research Group, Department of Biochemistry and Molecular Biology, University of British Columbia, Vancouver V6T 1Z3, Canada; Division of BioTherapeutics, Leiden Academic Centre for Drug Research, Leiden University, Leiden 2333 CC, The Netherlands; BioNanoPatterning, Department of Cell and Chemical Biology, Leiden University Medical Center, Leiden 2333 RC, The Netherlands; Materials and Interface Chemistry, Department of Chemical Engineering and Chemistry, Eindhoven University of Technology, Eindhoven 5600 MB, The Netherlands; Department of Biochemistry, Institute of Molecular Life Sciences, Radboud University Medical Center, Nijmegen 6500 HB, The Netherlands; Department of Biochemistry, University of Nebraska, Lincoln, NE 68588, USA; NanoMedicines Research Group, Department of Biochemistry and Molecular Biology, University of British Columbia, Vancouver V6T 1Z3, Canada; NanoMedicines Innovation Network (NMIN), University of British Columbia, Vancouver V6T 1Z3, Canada; NanoVation Therapeutics Inc., 2405 Wesbrook Mall 4th Floor, Vancouver V6T 1Z3, Canada; Supramolecular and Biomaterials Chemistry, Leiden Institute of Chemistry, Leiden University, Leiden 2333 CC, The Netherlands; NanoMedicines Research Group, Department of Biochemistry and Molecular Biology, University of British Columbia, Vancouver V6T 1Z3, Canada; NanoMedicines Innovation Network (NMIN), University of British Columbia, Vancouver V6T 1Z3, Canada; NanoVation Therapeutics Inc., 2405 Wesbrook Mall 4th Floor, Vancouver V6T 1Z3, Canada; Supramolecular and Biomaterials Chemistry, Leiden Institute of Chemistry, Leiden University, Leiden 2333 CC, The Netherlands

**Keywords:** embryonic zebrafish, lipid nanoparticles, mRNA delivery, reticuloendothelial system, stabilin-2

## Abstract

Lipid nanoparticles (LNPs) are the leading nonviral technologies for the delivery of exogenous RNA to target cells in vivo. As systemic delivery platforms, these technologies are exemplified by Onpattro, an approved LNP-based RNA interference therapy, administered intravenously and targeted to parenchymal liver cells. The discovery of systemically administered LNP technologies capable of preferential RNA delivery beyond hepatocytes has, however, proven more challenging. Here, preceded by comprehensive mechanistic understanding of in vivo nanoparticle biodistribution and bodily clearance, an LNP-based messenger RNA (mRNA) delivery platform is rationally designed to preferentially target the hepatic reticuloendothelial system (RES). Evaluated in embryonic zebrafish, validated in mice, and directly compared to LNP–mRNA systems based on the lipid composition of Onpattro, RES-targeted LNPs significantly enhance mRNA expression both globally within the liver and specifically within hepatic RES cell types. Hepatic RES targeting requires just a single lipid change within the formulation of Onpattro to switch LNP surface charge from neutral to anionic. This technology not only provides new opportunities to treat liver-specific and systemic diseases in which RES cell types play a key role but, more importantly, exemplifies that rational design of advanced RNA therapies must be preceded by a robust understanding of the dominant nano–biointeractions involved.

## 1. Introduction

RNA therapy relies on cytosolic delivery of exogenous (therapeutic) RNA molecules, e.g., messenger RNA (mRNA), small interefering RNA (siRNA), or microRNA (miRNA), to gain precise control of gene expression within target cells.^[1,2]^ This requires delivery systems to protect, transport, and deliver highly charged, immunogenic, and membrane impermeable RNA payloads within target cells and tissues in the body. To this end, lipid nanoparticles (LNPs) have emerged as the leading nonviral RNA delivery system for systemic in vivo application.^[3-5]^ These technologies are exemplified by Onpattro, a clinically approved LNP-based RNA interference therapy, administered intravenously (i.v.) and used to treat polyneuropathies resulting from transthyretin-mediated amyloidosis (hereditary transthyretin amyloidosis (hATTR)).^[6,7]^ Onpattro functions by transiently silencing transthyretin expression specifically within hepatocytes through siRNA delivery.^[7]^ Hepatocyte targeting is mediated through the adsorption of soluble apolipoprotein E (apoE) onto the surface of a circulating LNP.^[8,9]^ Once bound, apoE promotes LNP binding to the low density lipoprotein receptor (LDLr)^[10]^ that is heavily expressed on the sinusoidal surface of hepatocytes. ApoE–LDLr binding leads to LNP endocytosis and consequent cytosolic siRNA delivery. Cytosolic siRNA release is enhanced by the protonation of ionizable (cationic) lipids within the endosome and subsequent disruption of the endosomal membrane.^[11]^

Following systemic administration, harnessing apoE-mediated LNP specificity for the delivery of RNA therapeutics (siRNA or mRNA) to hepatocytes is relatively common.^[5,10,12-15]^ However, expanding the scope of LNP-based gene therapies to other hepatic cell types (or nonhepatic cells), and therefore gain access to many tissue-/cell-specific diseases, has so far proven more challenging. To meet this challenge, empirical screening of LNPs has revealed formulations that preferentially target extrahepatic tissues (e.g., bone marrow)^[16,17]^ and cells (e.g., T-cells),^[18,19]^ as well as individual hepatic (e.g., liver endothelial) cell types.^[20,21]^ However, while these empirical discoveries have enriched our understanding of the structure–activity land-scape of LNP technologies, they have not revealed the biological mechanisms underpinning LNP transport and preferential cellular uptake in vivo. This knowledge is fundamental for rational design and should be the foundation of any future discovery efforts toward LNP–RNA therapies with target cell specificity beyond hepatocytes.^[5,22]^

Besides hepatocytes (comprising ≈80% liver volume), the liver is composed of nonparenchymal liver cells, including Kupffer cells (KCs) and liver sinusoidal endothelial cells (LSECs).^[23]^ Hepatic blood vessels, or sinusoids, connecting the hepatic artery and portal vein to the central vein, are primarily composed of LSECs (≈70%) and KCs (≈20%).^[24,25]^ Together, these two cell types make up the hepatic reticuloendothelial system (RES) whose primary role is to maintain blood homeostasis through the scavenging of macromolecular waste and pathogens from blood.^[26,27]^ LSECs, in particular, are specialized scavenger endothelial cells (SECs) and have one of the highest endocytic activities of any cell type in the body.^[28]^ These cells are responsible for the clearance of endogenous macromolecules, such as oxidized low-density lipoprotein and hyaluronic acid,^[27,29,30]^ as well as blood-borne pathogens.^[31,32]^ In large part, LSEC clearance of macromolecular waste and pathogens is mediated through an array of scavenger receptors (e.g., hyaluronan and stabilin receptors), expressed on the luminal membrane of LSECs.^[33-35]^ As a therapeutic target, LSECs play a crucial role in liver homeostasis, regeneration following acute injury, and in the pathogenesis of various liver diseases, including cirrhosis and liver cancer.^[28,36]^ Additionally, as antigen presenting cells, LSECs are key regulators of hepatic adaptive immunity and systemic immunotolerance, and are therefore promising immunotherapy targets.^[37]^

Guided by a mechanistic understanding of the systemic clearance of i.v. administered anionic nanoparticles by hepatic RES cell types,^[38]^ here, we rationally design anionic LNPs to preferentially target and transfect the hepatic RES, i.e., scavenger receptor LNPs (srLNPs). This required just a single lipid compositional change within the formulation of Onpattro. Using the embryonic zebrafish (*Danio rerio*) as a convenient, accurate, and cost-effective in vivo model,^[39]^ we qualitatively describe LNP biodistribution, mRNA delivery, and expression of an exogenous fluorescent protein in vivo, at cellular resolution and in real time, focusing particularly on relative LNP uptake and mRNA expression within SECs, macrophages, and hepatocytes of the embryo. Furthermore, we confirm that scavenger receptors, stabilin-1 and −2, mediated uptake of anionic LNPs by SECs. Finally, we validate preferential LNP-mediated mRNA transfection of the hepatic RES in mice and demonstrate the critical importance of stabilin-2 for anionic LNP uptake and processing within mammalian LSECs.

## 2. Results

### 2.1. Design and Characterization of Anionic srLNPs

Previously, we have shown that i.v. administered, anionic nanoparticles (ranging in size from 10 to 150 nm and spanning a diverse range of chemistries) are rapidly and extensively cleared from circulation by SECs within the posterior cardinal vein (PCV), caudal hematopoietic tissue (CHT), and caudal vein (CV) of a two-day old zebrafish embryo.^[38]^ In teleost fish (i.e., zebrafish), and other aquatic vertebrates, SECs are not located primarily in the liver (as for LSECs in mammals), but reside in various other organs including scavenging (venous) blood vessels.^[40]^ Mechanistically, anionic nanoparticle recognition and uptake by SECs is mediated by the scavenger receptors, stabilin-1 (*stab1*) and stabilin-2 (*stab2*).^[38]^ Stabilin-1 and −2 are strongly expressed by LSECs in the mammalian liver^[29,41]^ and i.v. injection of anionic liposomes in 6–8 week old mice resulted in extensive anionic nanoparticle uptake within these cell types.^[38]^ In addition to SECs, anionic nanoparticles are also scavenged by blood resident macrophages, both within the CHT of the embryonic zebrafish and within the mouse liver (i.e., within KCs).^[38,42]^ Together, these observations indicate that the embryonic zebrafish can be used to qualitatively predict in vivo nanoparticle interactions with mammalian RES cell types.

**Figure 1.**
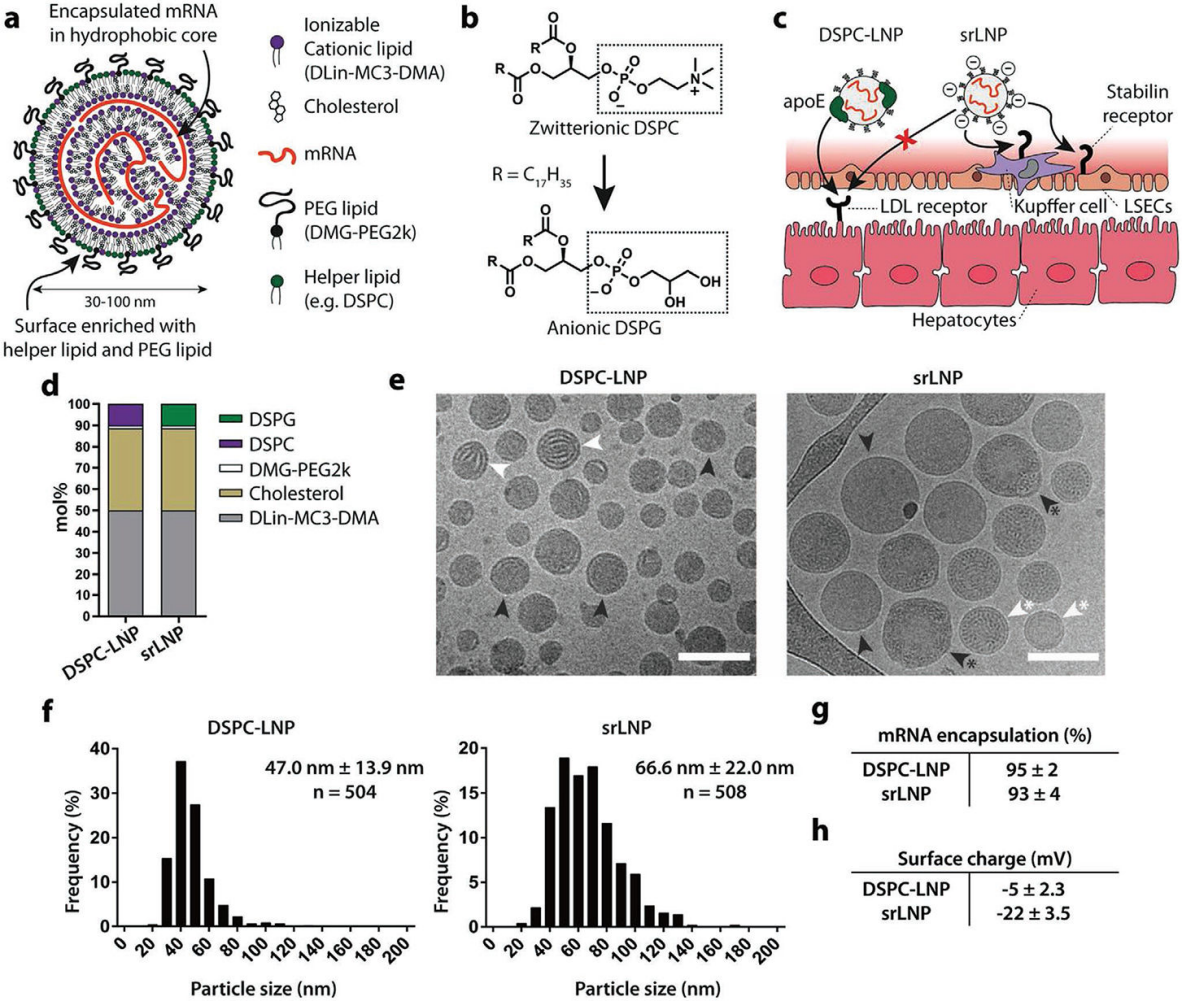
Design and characterization of srLNPs. a) Schematic of the structural organization of a LNP containing mRNA, as described previously.^[44]^ Helper phospholipids (typically incorporated at 10 mol%) are enriched at the LNP surface. b,c) Within the liver sinusoids, switching of the helper phospholipid from zwitterionic DSPC (as in Onpattro) to anionic DSPG created anionic srLNPs that are directed to the hepatic RES, via stabilin-receptor-mediated recognition and uptake in LSECs. srLNP uptake within hepatic RES cells is further enhanced by the inhibition of apoE–LDLr interactions mediated by anionic phospholipids (e.g., DSPG).^[45]^ The mechanism(s) of recognition and uptake of srLNPs by blood resident macrophages (i.e., KCs) are not fully known. d) Lipid composition of DSPC–LNPs (i.e., Onpattro) and srLNPs. e) Cryo-EM images of DSPC–LNPs and srLNPs (entrapping capped mRNA–*eCFP*) showing solid lipid nanoparticle structures. Scale bars: 100 nm. Internal structures indicated with arrows: lamellar (white), amorphous (black), polymorphous (black*), and unilamellar (white*). f) Size distribution of DSPC–LNPs and srLNPs, as determined by cryo-EM. The values derived from the frequency distribution graphs represent the mean ± standard deviation (s.d.). g) mRNA encapsulation efficiency within DSPC–LNPs and srLNPs, as determined by RiboGreen assay. h) Surface charge of DSPC–LNPs and srLNPs, as determined by zeta potential measurements. See Table S1 in the Supporting Information for full biophysical characterization of all formulations used in this study.

Here, we rationally designed an anionic LNP system for preferential genetic manipulation in hepatic RES cells. In general, LNPs consist of five structural components (four lipid reagents and an oligonucleotide payload) that self-assemble to form discrete nanostructures ranging from ≈30 to ≈150 nm in size (Figure 1a).^[43]^ The “hydrophobic” core of a LNP is rich in ionizable lipids (e.g., heptatriaconta-6,9,28,31-tetraen-19-yl 4-(dimethylamino)butanoate, DLin-MC3-DMA; 50 mol%*) [asterix denotes in the case of Onpattro]), cholesterol (38.5 mol%*), and an oligonucleotide payload. By contrast, the LNP surface (i.e., lipid–water interface) is rich in helper phospholipids (e.g., 1,2-distearoyl-*sn*-glycero-3-phosphocholine, DSPC; 10 mol%*) and lipid–polyethylene glycol (PEG) conjugates (e.g., 1,2-dimyristoyl-*rac*-glycero-3-methoxypolyethylene glycol-2000, DMG-PEG2k; 1.5 mol%*).^[44]^ We therefore hypothesized that by switching the helper phospholipid of Onpattro, from zwitterionic DSPC to its closest structural, but anionic, analog, 1,2-distearoyl-*sn*-glycero-3-phosphoglycerol (DSPG), we would render a LNP surface anionic while minimally disrupting LNP global structure. An anionic surface charge would, in turn, redirect LNP targeting and functional RNA delivery from hepatocytes to the hepatic RES through exploitation of a stabilin-mediated pathway of LNP recognition and uptake in LSECs while simultaneously inhibiting hepatocyte apoE–LDLr interactions (Figure 1b,c).^[45]^ Hereafter, we refer to DSPG-containing LNPs as srLNPs and LNPs based on the lipid composition of Onpattro as DSPC–LNPs (Figure 1d). LNP formulations with an effective anionic surface charge of ≤−15 mV have not been previously reported. In all cases, a nitrogen to phosphate (N:P) ratio of 6:1 was used, as is typical for larger nucleic acid payloads.^[46]^

Following microfluidic assembly, cryo-electron microscopy (cryo-EM) revealed LNPs with a typical electron-dense core structure (Figure 1e).^[47-50]^ Within DSPC–LNPs (47.0 ± 13.9 nm), both amorphous and lamellar core structures were present, whereas the core structure of srLNPs (66.6 ± 22.0 nm) contained a mixture of amorphous, unilamellar, and polymorphic structures, as has been previously reported for LNP–mRNA systems.^[51,52]^ Particle sizes of both DSPC–LNPs and srLNPs (determined through cryo-EM image analysis) were comparable to the number-weighted average determined by dynamic light scattering (Figure 1f and Table S1 (Supporting Information)) and both formulations were well below the size threshold considered favorable for size-dependent phagocytosis,^[53,54]^ as well as for unhindered passage through the fenestrae (180 ± 41 nm; murine) of the liver endothelium (i.e., unrestricted access to hepatocytes).^[55]^ In all cases, mRNA encapsulation efficiencies were >95% (Figure 1g). Crucially, however, srLNPs possessed a significantly more anionic (*ζ*-potential ≈ −20 mV) surface charge compared to DSPC–LNPs (*ζ*-potential ≈ −5 mV), indicative of DSPG exposed at the lipid–water interface (Figure 1h). For detailed biophysical characterization (i.e., size, surface charge, encapsulation efficiencies) of all formulations used in this study, please refer to Table S1 (Supporting Information).

### 2.2. Biodistribution of LNPs in Embryonic Zebrafish

**Figure 2.**
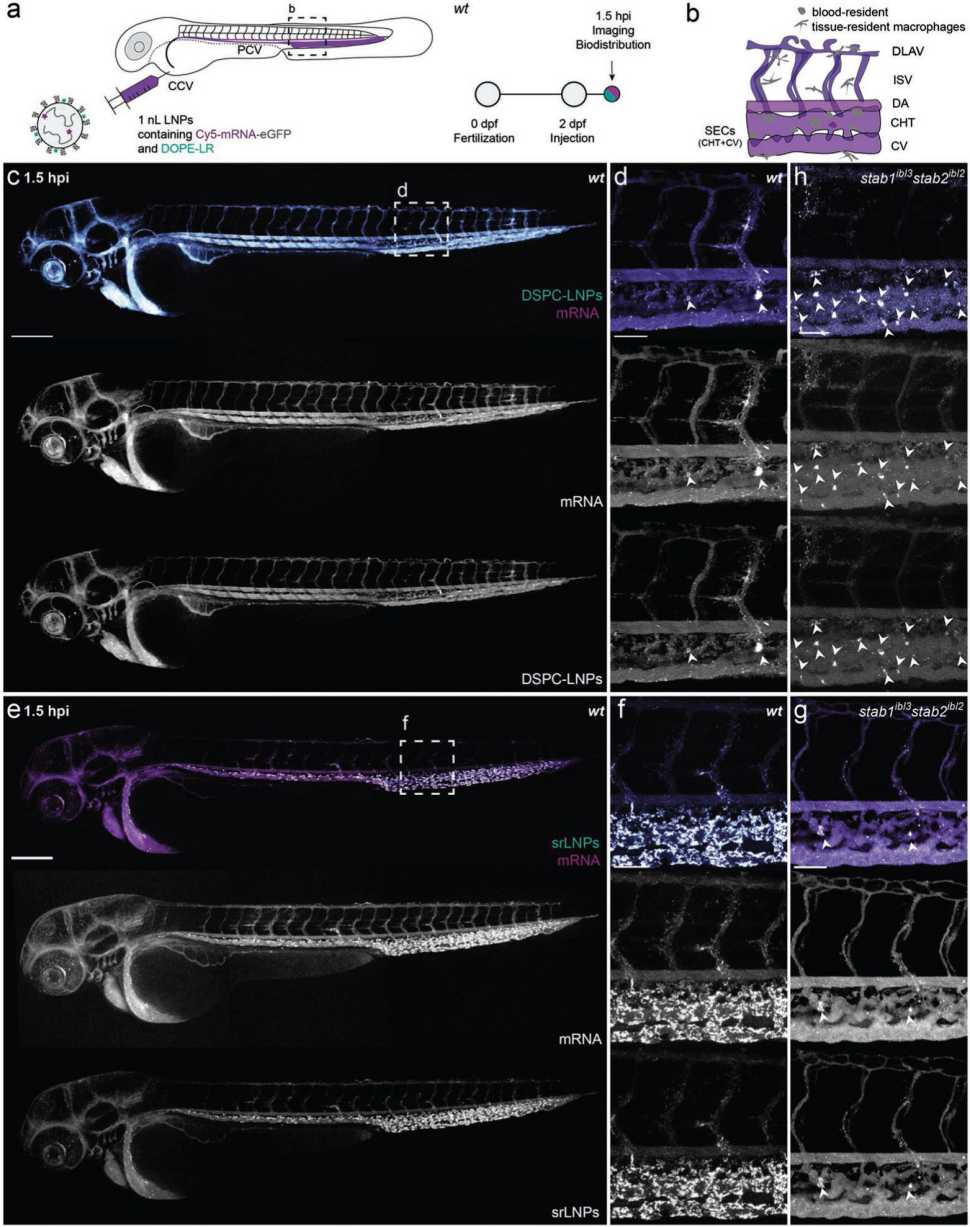
Biodistribution of DSPC–LNPs and srLNPs in two-day old embryonic zebrafish at 1.5 hpi. a) Schematic showing the site of LNP injection (i.v.) within embryonic zebrafish (2 dpf) and imaging timeframe. LNPs contained DOPE-LR (cyan, 0.2 mol%) as fluorescent lipid probe and Cy5-labeled *eGFP* mRNA (magenta) as fluorescent mRNA probe. Injected dose: ≈10 × 10^−3^ m lipid, ≈0.2 mg kg^−1^ mRNA. Injection volume: 1 nL. Major venous blood vessels: CCV: common cardinal vein; PCV: posterior cardinal vein. b) Tissue level schematic of a dorsal region of the embryo containing scavenging cell types (i.e., SECs and blood resident macrophages). Blood vessels: DA: dorsal aorta, CHT: caudal hematopoietic tissue; CV: caudal vein; ISV: intersegmental vessel; DLAV: dorsal longitudinal anastomotic vessel. c,d) Whole embryo (10× magnification) and tissue level (40× magnification) views of DSPC–LNP biodistribution within wild-type (AB/TL) embryonic zebrafish (2 dpf) at 1.5 hpi. DSPC–LNPs were mostly freely circulating, confined to, and distributed throughout, the vasculature of the embryo. Low level phagocytotic uptake within blood resident macrophages is highlighted with white arrowheads. e,f) Whole embryo (10× magnification) and tissue level (40× magnification) views of srLNP biodistribution within wild-type (AB/TL) embryonic zebrafish (2 dpf) at 1.5 hpi. srLNPs were mainly associated with SECs within the PCV, CHT, and CV of the embryo and were largely removed from circulation at 1.5 hpi. Phagocytotic uptake of both DSPC–LNPs and srLNPs within blood resident macrophages at 1.5 hpi was confirmed by analogous LNP injections in transgenic *mpeg*:*mCherry* zebrafish embryos, stably expressing *mCherry* within all macrophages (see Figure S1 in the Supporting Information). g) Tissue level (40× magnification) view of srLNP biodistribution within *stab1*^*−/−*^/*stab2*^*−/−*^ mutant zebrafish embryos^[56]^ at 1.5 hpi. Within stabilin KOs, srLNPs were now mostly freely circulating, with low level phagocytotic uptake within blood resident macrophages highlighted by white arrowheads. h) Tissue level (40× magnification) view of DSPC–LNP biodistribution within *stab1*^*−/−*^/*stab2*^*−/−*^ mutant zebrafish embryos^[56]^ at 1.5 hpi. Within stabilin KOs, DSPC–LNPs remain mostly freely circulating, with low level phagocytotic uptake within blood resident macrophages highlighted by white arrowheads. For whole embryo images of LNP biodistribution within *stab1^−/−^*/*stab2*^*−/−*^ mutant zebrafish embryos^[56]^ at 1.5 hpi, please see Figure S2 in the Supporting Information. Scale bars: 200 μm (whole embryo) and 50 μm (tissue level).

To assess LNP in vivo biodistribution, DSPC–LNPs and srLNPs containing a fluorescent lipid probe (1,2-dioleoyl-sn-glycero-3-phosphoethanolamine-N-(lissamine rhodamine B sulfonyl (DOPE-LR), 0.2 mol%)) and encapsulating fluorescently tagged mRNA (capped and Cy5-labeled), were injected (i.v., ≈10 × 10^−3^ m lipid, ≈0.2 mg kg^−1^ mRNA) in wild-type zebrafish embryos at two days postfertilization (dpf) (Figure 2a). Confocal imaging of entire live embryos, as well as high resolution, tissue level views to include key scavenging cell types of the embryo within the CV and CHT (Figure 2b), revealed distinct biodistribution patterns for both LNP–mRNA formulations at 1.5 h postinjection (hpi) (Figure 2c-f). In the case of DSPC–LNPs, particles were mostly freely circulating, with both lipid and mRNA confined to, and homogenously distributed throughout, the vasculature of the embryo (Figure 2c,d). In addition, a small fraction of DSPC–LNPs accumulated within blood-resident macrophages of the CHT, indicative of low-level recognition and uptake by the RES (white arrowheads, Figure 2d; confirmed in Tg(*mpeg*:*mCherry*) embryos, Figure S1a-d, Supporting Information). In the case of srLNPs, the majority of injected particles were cleared from circulation at 1.5 hpi, with highly selective accumulation observed within SECs and blood-resident macrophages of the PCV, CHT, and CV (Figure 2e,f; macrophage uptake confirmed in Tg(*mpeg*:*mCherry*) embryos and Figure S1e-g (Supporting Information)).

This selective accumulation of srLNPs within scavenging (venous) blood vessels of the embryonic zebrafish closely resembled that previously observed for anionic liposomes, polymersomes, and inorganic nanoparticles, in which nanoparticle uptake within SECs was mediated by stabilin scavenger receptors.^[38]^ To therefore confirm stabilin-mediated uptake, srLNPs were injected (i.v.) in established *stab1*^*−/−*^/*stab2*^*−/−*^ double knockout (KO) zebrafish embryos (2 dpf).^[56]^ Within these mutant embryos, srLNPs predominantly remained in circulation at 1.5 hpi with a small fraction accumulating within blood-resident macrophages of the CHT (Figure 2g and Figure S2 (Supporting Information) for whole embryo images). This confirmed that srLNPs selectively accumulate within RES cell types of the embryonic zebrafish and that recognition and uptake of srLNPs within SECs, but not macrophages, are exclusively mediated by stabilin receptors. Analogous injections of DSPC–LNPs within double KO (DKO) embryos did not alter DSPC–LNP biodistribution, with the majority of DSPC–LNPs remaining in circulation (Figure 2h and Figure S2 (Supporting Information) for whole embryo images). In all cases, both lipid and mRNA fluorescent probes appear fully colocalized at 1.5 hpi, indicating that mRNA remained stably entrapped within the core of both DSPC and srLNPs in circulation, as well as during cellular recognition and (early) cellular uptake.

### 2.3. LNP-Mediated mRNA Delivery and Expression in Embryonic Zebrafish

**Figure 3.**
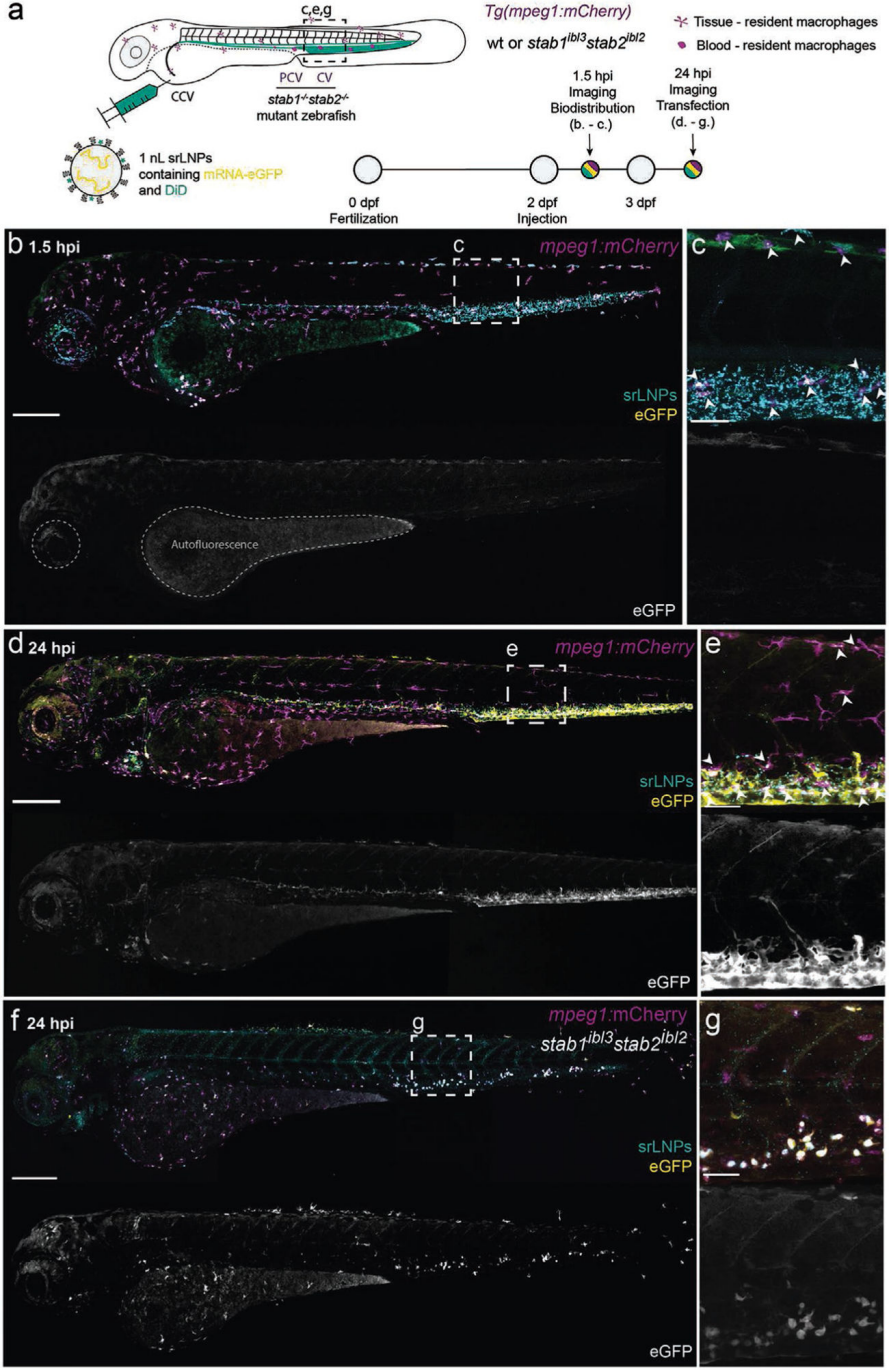
srLNP biodistribution, *eGFP*–mRNA delivery, and *eGFP* expression within *mpeg1:mCherry* transgenic zebrafish embryos at 1.5 and 24 hpi. a) Schematic showing the site of srLNP injection (i.v.) within embryonic zebrafish (2 dpf) and imaging timeframe. srLNPs contained DiD (Cy5, 0.1 mol%) as fluorescent lipid probe and unlabeled, *eGFP* mRNA (capped) payload. Injected dose: ≈10 × 10^−3^ m lipid, ≈0.2 mg kg^−1^ mRNA. Injection volume: 1 nL. Transgenic Tg(*mpeg1:mCherry*) zebrafish embryos stably express *mCherry* (magenta) within all macrophages. b,c) Whole embryo (10× magnification) and tissue level (40× magnification) views of srLNP biodistribution and *eGFP* expression within the embryonic zebrafish at 1.5 hpi. At this timepoint, srLNPs were mainly associated with SECs and blood resident macrophages (white arrowheads) within the PCV, CHT, and CV of the embryo and largely removed from circulation. Low-level autofluorescence in the GFP channel is highlighted within the yolk sac and pigment cells of the embryo. d,e) Whole embryo and tissue level views of srLNP biodistribution and *eGFP* expression within the embryonic zebrafish at 24 hpi. At this timepoint, srLNPs remain associated with SECs and blood resident macrophages (white arrowheads) within the PCV, CHT, and CV of the embryo. However, intense *eGFP* expression was now observed specifically within the PCV, CHT, and CV confirming successful cytosolic delivery and translation of functional *eGFP* mRNA within SECs and blood resident macrophages. f,g) Whole embryo and tissue level views of srLNP biodistribution and *eGFP* expression within *stab1*^*−/−*^/*stab2*^*−/−*^ mutant embryos at 24 hpi. In these mutant embryos, *eGFP* expression was predominantly observed in blood resident macrophages of the CHT, confirming the requirement of stabilin receptors for srLNP-mediated mRNA expression within SECs. Scale bars: 200 μm (whole embryo) and 50 μm (tissue level).

To assess LNP-mediated delivery of functional mRNA within the embryonic zebrafish, we switched to unlabeled *eGFP* mRNA (capped, Figure 3a), as we consistently observed low mRNA expression levels using Cy5-labeled *eGFP* (capped) mRNA payloads. This alteration did not significantly change the structure, surface charge, or mRNA encapsulation efficiency of LNPs (see Table S1 in the Supporting Information). At 1.5 hpi, srLNPs (≈10 × 10^−3^ m lipid, ≈0.2 mg kg^−1^ mRNA) again associated with SECs and blood-resident macrophages within the PCV, CHT, and CV of the embryonic zebrafish (Figure 3b,c). Given the >2 h timeframe for mRNA delivery, expression and maturation of *eGFP*,^[57,58]^ low level green fluorescence observed at 1.5 hpi, within the yolk sac and iridophores (pigment cells) of the embryo, is attributed to embryo autofluorescence in the GFP channel.^[59]^ At 24 hpi, however, intense *eGFP* fluorescence was observed specifically within SECs and blood-resident macrophages of the embryo (Figure 3d,e). This is consistent with the timings reported for *eGFP*–mRNA delivery and expression using analogous lipid-based delivery systems, whereby the onset of *eGFP* maturation and fluorescence (in vitro) occurs 2–7 h postincubation and expression levels (fluorescence intensity) continually increase up to 24 h post-treatment.^[57,60]^ Within *stab1*^*−/−*^/*stab2*^*−/−*^ mutant embryos, srLNP-mediated *eGFP* expression at 24 hpi was observed within blood-resident macrophages, but not SECs, confirming that macrophage uptake of srLNPs, as for other anionic nanoparticles, is not exclusively dependent on stabilin receptors (Figure 3f,g).

In the case of srLNPs, the observed pattern of *eGFP* expression, within SECs and macrophages of the CHT and CV, mirrored srLNP biodistribution at 1.5 hpi (Figure 2e,f) and confirmed successful transport, uptake, and cytosolic delivery of functional mRNA within these cells. This is particularly remarkable given SECs have one of the highest endo-/lysosomal activities of any cell type^[30,35]^ and are primed to degrade fragile RNA molecules. Endosomal escape and cytosolic delivery of RNA is recognized as one of the major obstacles in the development of effective RNA therapies,^[61]^ with <2% of internalized siRNA (complexed within LNPs based on the lipid composition of Onpattro) reaching the cytoplasm of HeLa cells (in vitro) and hepatocytes (in vivo).^[62,63]^ Indeed, the acute extent of mRNA degradation within SECs (as well as potential mRNA degradation in circulation), was partly confirmed by injection (i.v.) of free *eGFP*–mRNA (capped; both Cy5-labeled and unlabeled) within the zebrafish embryo. This resulted in no significant expression of *eGFP* within SECs at 24 hpi despite extensive accumulation within these cells at 1.5 hpi, presumably via scavenger-receptor-mediated uptake of circulating, polyanionic RNA (Figure S3, Supporting Information).^[64]^

In the case of DSPC–LNP-mediated mRNA delivery (≈10 × 10^−3^ m lipid, ≈0.2 mg kg^−1^ mRNA), widespread *eGFP* fluorescence was observed throughout the two-day old embryo at 24 hpi (Figure S4, Supporting Information). Combined with the evident lack of cellular accumulation at 1.5 hpi (Figure 2c,d), this suggested that LNPs based on the lipid composition of Onpattro are subject to low-level, nonspecific cellular uptake with resultant mRNA expression across a broad range of cell types, including SECs and blood-resident macrophages. Importantly, however, the two-day old embryonic zebrafish lacks a functional liver system.^[65,66]^ To assess potentially important and/or competitive (apoE-mediated) pathways of LNP recognition and uptake within functional hepatocytes, therefore, it was necessary to switch to LNP injections in older zebrafish embryos.

### 2.4. Hepatocyte Targeting and mRNA Expression in Older Zebrafish Embryos

From ≈55 h postfertilization (hpf), the liver of the embryonic zebrafish undergoes a dramatic growth phase. New intrahepatic blood vessels are formed, with blood circulation detected from 72 hpf,^[67]^ and the localized expression of key hepatocyte markers, including transferrin^[68]^ and liver fatty acid binding protein (*L-FABP*),^[69]^ evidently marking maturation of functional hepatocytes. During this growth phase, anatomical features characteristic of the mammalian liver, and necessary for hepatic processing of lipid nanoparticles, also emerge, including a space of Disse,^[70]^ the likely presence of a fenestrated endothelium^[71]^ and a functional biliary network (connected to the blood vasculature via hepatocytes).^[65]^ At this developmental stage, the embryonic zebrafish also possesses a conserved repertoire of lipid transport proteins,^[72,73]^ including apoE and lipoprotein receptors, (e.g., LDLr).^[74,75]^ In the case of apoE, zebrafish expresses two isoforms (apoEa and apoEb) which, despite relatively low overall sequence similarity (28–51% homology), have highly conserved LDLr/LRP binding domains (residues 122–131) when compared to human apoE (residues 141–150). Altogether, this functionally conserved array of lipid processing pathways, proteins, and cells has led to the use of the embryonic zebrafish as in vivo model to study various aspects of endogenous (apoE-mediated) lipid transport and metabolism,^[76,77]^ both in the diseased and healthy states.^[72,74,75,78]^ Altogether, these features and reports indicate a potentially valuable role for zebrafish embryos as predictive in vivo screening platforms for studying both the clearance and metabolism of lipid-based nanomaterials.

**Figure 4.**
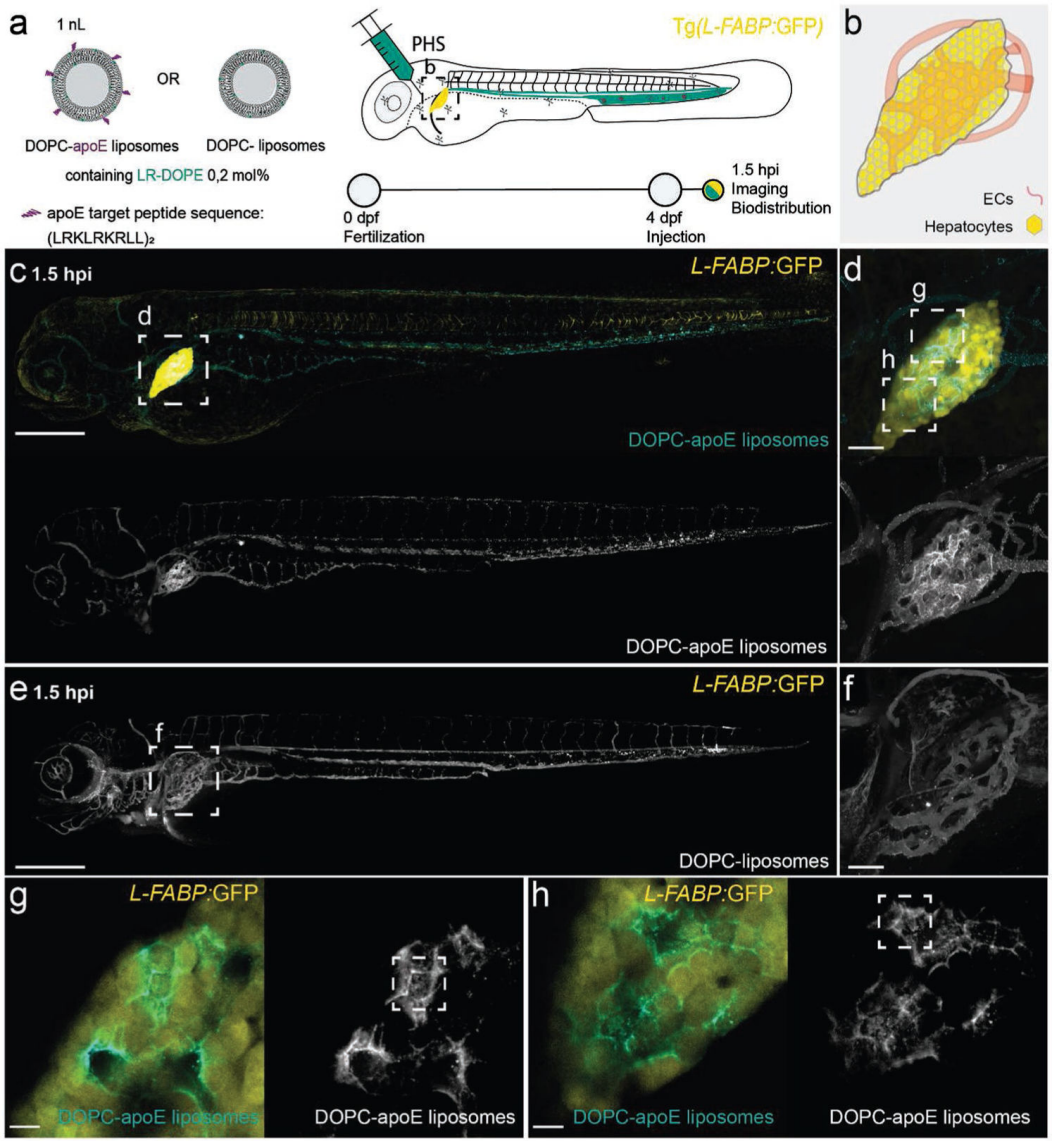
Biodistribution of apoE-targeted liposomes in four-day old zebrafish embryos. a) Schematic showing the site of apoE-targeted DOPC or (nontargeted) DOPC liposome injection (i.v.) within 4-day old embryonic zebrafish and imaging timeframe. Liposomes contained 0.2 mol% DOPE–lissamine rhodamine as fluorescent lipid probe (cyan). Injected dose: ≈5 × 10^−3^ m lipid, ≈5 mol% apoE target ligand (amino acid primary sequence: (LRKLRKRLL)_2_), injection volume: 1 nL. PHS: primary head sinus. Transgenic Tg(*L-FABP*:*eGFP*) zebrafish embryos stably express *eGFP* (yellow) within all hepatocytes. b) Tissue level schematic of the embryonic liver at 4 dpf. c,d) Whole embryo (10× magnification) and tissue (liver) level (40× magnification) view of apoE-targeted DOPC liposome biodistribution within four-day old embryonic zebrafish at 1.5 hpi. At this timepoint, apoE-target DOPC liposomes clearly accumulated within the liver of the embryo. e,f) Whole embryo (10× magnification) and tissue (liver) level (40× magnification) views of DOPC liposome biodistribution within four-day old embryonic zebrafish at 1.5 hpi. At this timepoint, unmodified DOPC liposomes are predominantly freely circulating throughout the vasculature of the zebrafish embryo. g,h) Zoom in regions of liver of apoE-targeted DOPC liposome biodistribution. Stacks of 3 confocal slices (6 μm thickness) show diffuse liposome-associated fluorescence within hepatocytes (i.e., uptake) as well as clear delineation of the characteristic hexagonal morphology of hepatocytes (i.e., stockpiling within the space of Disse)—examples of both phenomena highlighted in white boxes. Scale bars: 200 μm (whole embryo), 50 μm (tissue level), and 10 μm (zoom).

To establish the embryonic zebrafish as an in vivo model for apoE-mediated targeting of lipid-based nanomedicines, we first administered apoE-targeted 1,2-dioleoyl-sn-glycero-3-phosphocholine (DOPC) liposomes (≈100 nm; Chol─NH─apoE peptide, 5 mol%, see the Supporting Information for synthesis and characterization) within 4-day old, Tg(*L-FABP*:*eGFP*) zebrafish embryos (Figure 4a,b). Nanoparticle-/macromolecule-conjugated apoE target peptides (amino acid sequence: (LRKLRKRLL)_2_; tandem-repeat LDLr target sequence (residues 141–150) of human apoE) have been previously shown to interact with LDLr, as well as the low-density lipoprotein-receptor-related proteins (LRPs), LRP1 and LRP2.^[79-81]^ Following i.v. administration, apoE-targeted DOPC liposomes clearly associated within the liver of a four day embryo (Figure 4c,d). By contrast, nontargeted DOPC liposomes remained freely circulating (Figure 4e,f). This confirmed that apoE-mediated pathways of nanoparticle recognition and uptake within the liver of a four-day old zebrafish are present, functional, and exploitable. Within the liver itself, apoE-targeted liposomes were not only taken up by hepatocytes but could be clearly observed delin-eating the characteristic hexagonal morphology of hepatocytes (Figure 4g,h). This apparent “stockpiling” of apoE-targeted liposomes within the space of Disse (i.e., associated with, but not yet taken up by, hepatocytes), was previously observed during the hepatic processing of albumin within zebrafish embryos,^[71]^ and may reflect extended (i.e., >1.5 hpi) timings of nanoparticle trafficking and processing within the liver.^[82]^ Overall, our observations, supported by the conserved repertoire of lipid transport and metabolism pathways, proteins, and cell types, strongly suggest that apoE-mediated mechanisms of LNP–hepatocyte targeting are present, functional, exploitable—and can be clearly distinguished by fluorescence confocal microscopy—within a four-day old zebrafish embryo.

**Figure 5.**
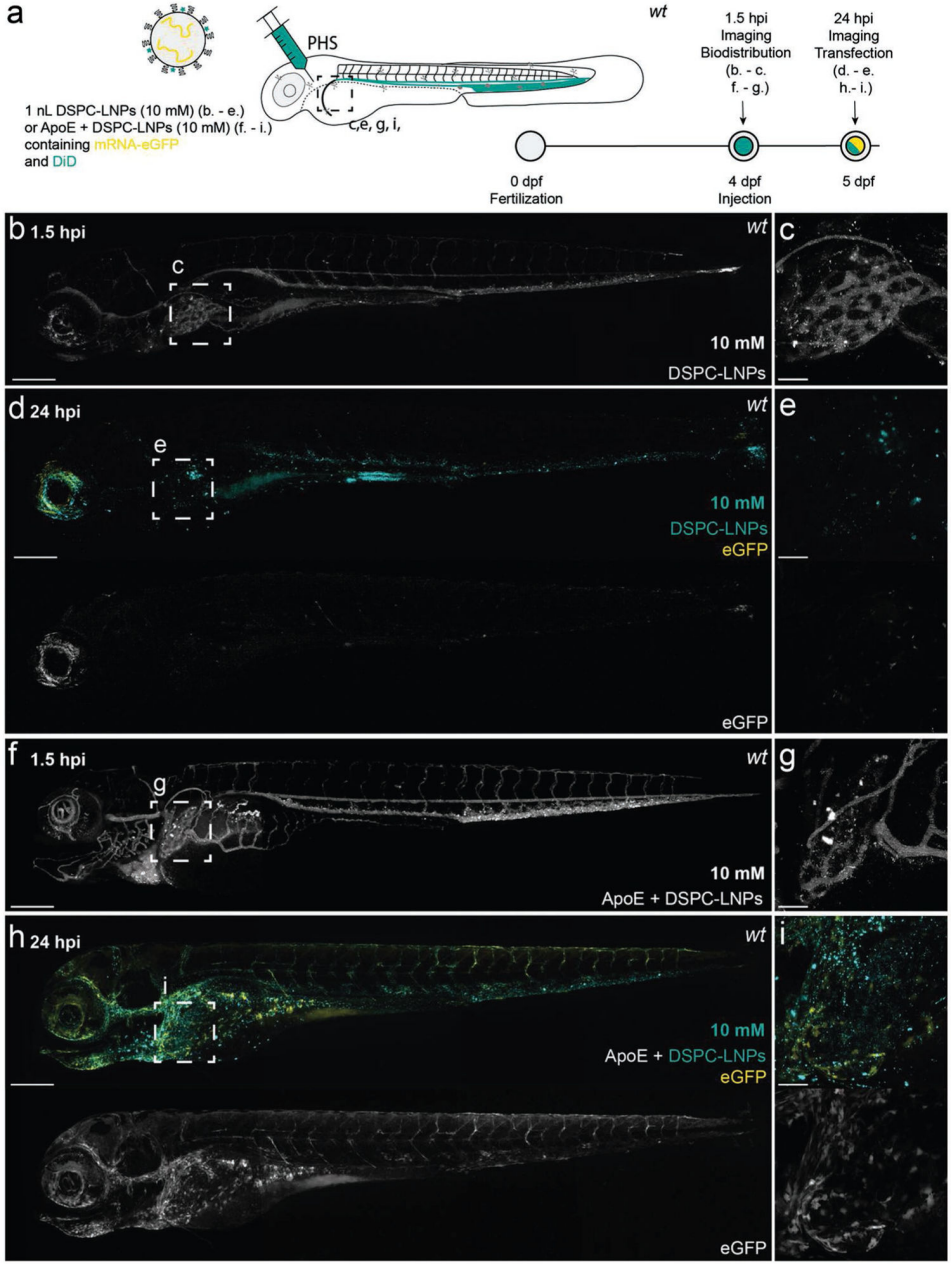
DSPC–LNP biodistribution and mRNA expression within, four-day old, wild-type (AB/TL) embryonic zebrafish, with and without preincubation with human apoE. a) Schematic showing the site of DSPC–LNP injection (i.v.) within embryonic zebrafish (4 dpf). DSPC–LNPs (10 × 10^−3^ m) contained DiD (0.1 mol%) as fluorescent lipid probe and unlabeled, *eGFP* mRNA (capped) payload after 1 h incubation with/without human apoE. Injection and imaging timeframe. Injection volume: 1 nL. PHS: primary head sinus. b,c) Whole embryo (10× magnification) and tissue level (liver region, 40× magnification) views of DSPC–LNP biodistribution at 1.5 hpi. Injected dose: ≈10 × 10^−3^ m lipid, ≈0.2 mg kg^−1^ mRNA. LNPs were mostly freely circulating with no significant accumulation in the liver at 1.5 hpi. Intense fluorescent punctae within the liver region are likely due to macrophage uptake. d,e) Whole embryo (10× magnification) and tissue level (liver region, 40× magnification) views of *eGFP* expression at 24 hpi. f,g) Whole embryo (10× magnification) and tissue level (liver region, 40× magnification) views of DSPC–LNP biodistribution, following preincubation (1 h) with apoE (5 mg μL^−1^; 1:1 v/v), at 1.5 hpi. Injected dose: ≈10 × 10^−3^ m lipid, ≈0.2 mg kg^−1^ mRNA. LNPs were mostly freely circulating with no significant accumulation in the liver observed at 1.5 hpi. Intense fluorescent punctae within the liver region are likely due to macrophage LNP uptake. h,i) Whole embryo (10× magnification) and tissue level (liver region, 40× magnification) views of *eGFP* expression at 24 hpi. In this case, a qualitative increase in liver-specific *eGFP* expression was observed. Scale bars: 200 μm (whole embryo) and 50 μm (tissue level).

To investigate potential apoE-mediated pathways of LNP recognition and uptake, we injected DSPC–LNPs (Figure 5a (≈10 × 10^−3^ m lipid) and Figure S5 (Supporting Information, ≈30 × 10^−3^ m lipid)) and srLNPs (Figure S6, Supporting Information, ≈30 × 10^−3^ m lipid) within 4-day old zebrafish embryos. In the case of anionic srLNPs (≈30 × 10^−3^ m, ≈0.6 mg kg^−1^ mRNA), LNPs once again associated with SECs and blood resident macrophages within the PCV, CV, and CHT at 1.5 hpi (Figure S6, Supporting Information), consistent with our observations in two-day old embryos (Figure 2e,f). Likewise, srLNP-mediated *eGFP* expression was largely restricted to RES cell types with no significant liver-specific mRNA expression observed (Figure S6, Supporting Information). This result indicated that stabilin-mediated mechanisms of srLNP recognition and uptake within SECs predominate over any potentially competitive apoE-mediated, LNP processing pathways within the embryonic zebrafish.

In the case of DSPC–LNPs (≈10 × 10^−3^ m, ≈0.2 mg kg^−1^ mRNA), we observed (very) low level and widespread *eGFP* expression throughout the embryo with no preferential hepatic accumulation of LNPs (at 1.5 hpi) or enhanced, liver-specific *eGFP* expression at 24 hpi (Figure 5b-e). This biodistribution and mRNA expression profiles were mirrored at higher DSPC–LNP dosages (≈30 × 10^−3^ m, ≈0.6 mg kg^−1^ mRNA), albeit at enhanced global levels of *eGFP* mRNA expression (Figure S6, Supporting Information). Preincubation (1 h) of DSPC–LNPs (≈10 × 10^−3^ m, ≈0.2 mg kg^−1^ mRNA) with human apoE (5 mg μL^−1^), however, did result in a qualitative increase in *eGFP* mRNA expression within the embryonic liver, despite no obvious enhancement of DSPC–LNP hepatocyte targeting at 1.5 hpi (Figure 5f-i). These results suggest that while DSPC–LNP-mediated mRNA expression within hepatocytes may be enhanced by apoE-mediated pathways of recognition and uptake, endogenous apoE-mediated pathways of LNP processing, at least in the zebrafish embryo, are relatively inefficient.

### 2.5. LNP-Mediated mRNA Delivery and Expression in Mice

**Figure 6.**
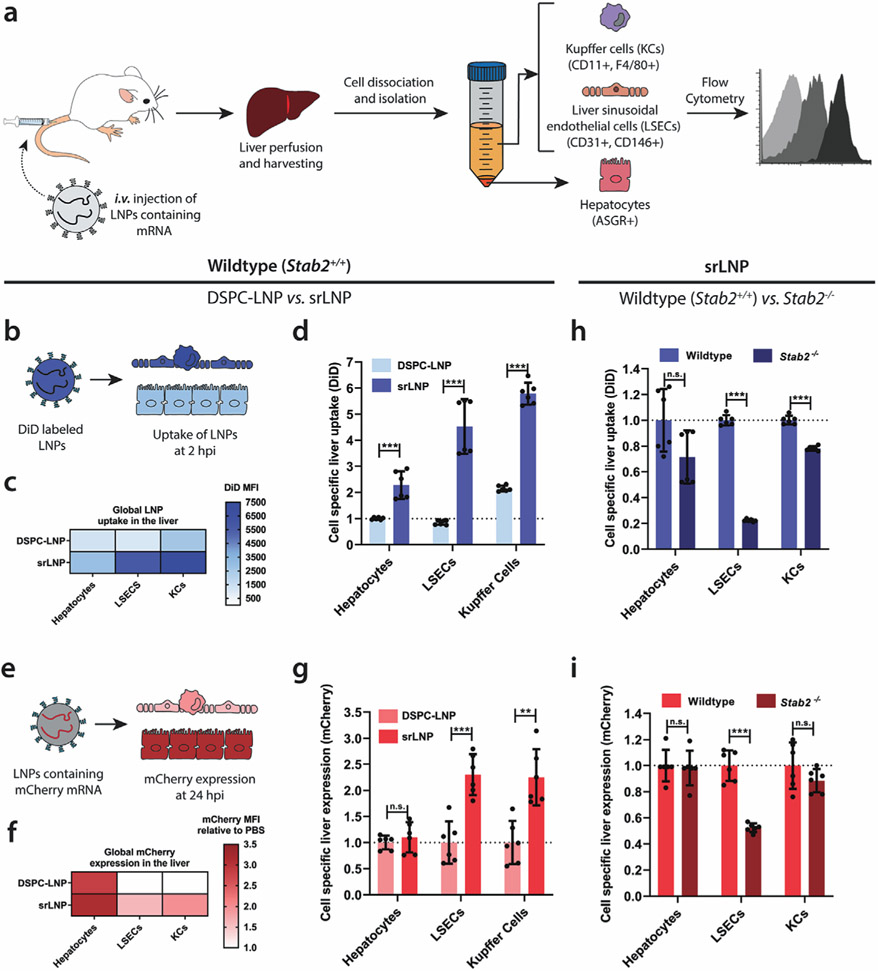
LNP uptake and functional mRNA delivery within different hepatic cell types following i.v. administration in mice. a) Schematic illustrating the procedure to isolate different hepatic cell types and determine LNP–mRNA targeting and functional mRNA delivery. Following intravenous LNP–mRNA injection (i.v.) the liver was perfused with collagenase IV, hepatic cells were isolated and stained with specific antibodies, and flow cytometry was used to analyze LNP uptake and gene expression. Specific antibody markers used to uniquely identify hepatocytes, LSECs and KCs, respectively, are defined in parentheses. b) For intrahepatic biodistribution studies, LNPs contained DiD (0.5 mol%) as fluorescent lipid probe. Cellular uptake of DSPC–LNP and srLNP was assessed following mouse sacrifice at 2 hpi. Injected dose: 42.75 mg kg^−1^ total lipid. c) Heatmap of global LNP uptake in the liver determined by absolute DiD fluorescence. srLNP demonstrated significantly enhanced LNP uptake within all hepatic cell types, and significant redirection to hepatic RES compared to DSPC–LNPs. d) Cell-specific liver uptake normalized to DSPC–LNP in liver hepatocytes. e) For gene expression experiments, LNPs contained capped, *mCherry*–mRNA. Functional mRNA delivery was assessed based on *mCherry* fluorescence levels following mouse sacrifice at 24 hpi. f) Heatmap of *mCherry* expression in different liver cell types following functional mRNA delivery using DSPC–LNP and srLNP. Injected dose: 0.25 mg kg^−1^ mRNA. g) Cell-specific *mCherry* expression normalized to DSPC–LNP for each cell type. h) Cell-specific liver uptake of srLNP in wild-type and mutant *stab2*^*−/−*^ KO mice, normalized to srLNP in wild-type for each cell type. i) Cell-specific liver expression of srLNP in wild-type and mutant *stab2*^*−/−*^ KO mice, normalized to srLNP in wild-type for each cell type. In all cases, *n* = 6; representing 3 separate liver tissue samples from 2 mice sorted into individual cell types. Bars and error bars in (d) and (g) represent mean ± s.d. The data were normalized to the average uptake and expression of DSPC–LNPs within each cell type. Statistical significance was evaluated using a two-tailed unpaired Student’s *t*-test. n.s. = not significant *p* > 0.01, * *p* < 0.01, ** *p* < 0.01, *** *p* < 0.001. Exact *p* values for (d): hepatocytes *p* = 0.000147, LSECs *p* = 6.20 × 10^−6^, KCs *p* = 1.65 × 10^−9^. Exact *p* values for (g): hepatocytes *p* = 0.464, LSECs *p* = 0.000215, KCs *p* = 0.00113. Exact *p* values for (h): hepatocytes *p* = 0.0531, LSECs *p* = 5.62 × 10^−13^, KCs *p* = 5.78 × 10^−8^. Exact *p* values for (i): hepatocytes *p* = 0.808, LSECs *p* = 2.33 × 10^−6^, KCs *p* = 0.188.

Next, we validated LNP biodistribution and LNP-mediated mRNA expression patterns in mice, focusing on cell-specific LNP distribution and mRNA expression within the murine liver, the largest RES organ in mammals. For all wild-type mouse experiments, LNP–mRNA formulations were injected (i.v.) in 8–10 week old C57BL/6 mice (Figure 6a). To assess LNP distribution and functional mRNA delivery within individual hepatic and nonhepatic (i.e., spleen and bone marrow) RES cell types, mice were anesthetized, a trans-cardiac collagenase perfusion performed, (parenchymal and nonparenchymal hepatic) cells separated, and individual cell types detected using cell-specific antibodies (see Figure S7 in the Supporting Information for representative flow cytometry density plots). To monitor LNP biodistribution across RES cell types and tissues, LNP–mRNA formulations, containing a nonexchangeable, fluorescent lipid probe (1,1-dioctadecyl-3,3,3,3-tetramethylindodicarbocyanine (DiD), 0.5 mol%), were administered (Figure 6b). Importantly, this allowed us to full decouple and independently assess cell-specific LNP targeting and resultant mRNA expression.

At 2 hpi, for both DSPC–LNPs and srLNPs (42.75 mg kg^−1^ total lipid), we observed extensive LNP accumulation within the mouse liver (Figure 6c,d) as compared to accumulation in other RES organs, namely bone marrow and spleen (Figure S8, Supporting Information), the latter being a smaller but highly efficient unit of the mononuclear phagocyte system.^[83]^ Notably, both LNP formulations distributed to all hepatic cell types, as has previously been described for LNP formulations based on Onpattro,^[82,84]^ however, srLNPs showed significantly enhanced uptake (*p* < 0.001) in all liver cell types (Figure 6c). Most strikingly, srLNPs yielded an approximately fivefold and an approximately threefold targeting enhancement to murine LSECs and KCs, respectively (Figure 6d). This confirmed that the incorporation of anionic DSPG into LNP–mRNA delivery systems not only enhanced liver tropism in general but led to a significant shift toward preferential LNP targeting and cellular uptake within hepatic RES cell types.

To confirm functional mRNA delivery to hepatic RES cells, LNPs entrapping capped *mCherry*–mRNA (0.25 mg kg^−1^ mRNA) were administered (Figure 6e). This dosage is in line with other systemically administered LNP–mRNA therapies, including those currently in clinical trials (e.g., NCT03829384).^[85]^ Following organ isolation and cell separation at 24 hpi, srLNPs yielded significantly enhanced mRNA delivery to hepatic RES cell types relative to DSPC–LNPs (*p* < 0.001) (Figure 6f,g). Indeed, DSPC–LNP-mediated *mCherry* expression in hepatic RES cell types was indiscernible above background. Within hepatocytes, and despite comparatively low targeting efficiency to these cells, both srLNPs and DSPC–LNPs expressed significantly higher amounts of *mCherry* than in hepatic RES cells (Figure 6f).

This apparent disparity between liver-cell-specific targeting and resultant mRNA expression levels can be explained by the very different physiologies and endogenous functions of hepatocytes versus hepatic RES cells. LSECs and KCs have very high endo-/phagocytic capacity (leading to significant mRNA degradation despite high LNP uptake), whereas hepatocytes have exceptionally high translational capacity (leading to significant mRNA expression despite comparably low LNP recognition and uptake).^[86]^ While it is common practice to use RNA expression/knockdown as an indirect (albeit therapeutically relevant) measurement of LNP targeting, our results clearly demonstrate that, particularly in the case of the liver, this indirect readout may be a very poor predictor of LNP targeting specificity. Furthermore, by decoupling targeting and mRNA expression, our results revealed the relatively inefficient targeting of Onpattro-like LNPs to hepatocytes in mammals. Corroborating our findings in the embryonic zebrafish (Figure 5), this result reaffirmed our believe that endogenous apoE-mediated LNP targeting of hepatocytes is a relatively inefficient, albeit significant, biological pathway of LNP recognition and uptake in both embryonic zebrafish and adult mammals.

Finally, to confirm a stabilin-mediated mechanism of hepatic RES targeting, srLNPs were administered (i.v.) within established *stab2*^*−/−*^ KO mice.^[87]^ Phenotypically normal *stab2*^*−/−*^ mice were specifically selected as *stab1*^*−/−*^/*stab2*^*−/−*^ DKO mice suffer from premature mortality and develop severe glomerular fibrosis.^[34]^ Therefore, we first confirmed the primary significance of stabilin-2 over stabilin-1 in the recognition and clearance of srLNPs, in single KO (*stab1*^*−/−*^ or *stab2*^*−/−*^) mutant zebrafish embryos (Figure S9, Supporting Information), as was expected.^[38]^ Following i.v. injection within *stab2*^*−/−*^ mice, srLNPs yielded an ≈80% reduction in srLNP targeting to LSECs and an ≈50% reduction in LSEC-specific mRNA expression (Figure 6h,i). By contrast, loss of stabilin-2 resulted in only a small targeting reduction to KCs (which expresses stabilin-2 but likely involves other receptors in LNP recognition and uptake) and had no significant effect on srLNP recognition and/or processing within hepatocytes (which do not express stabilin-2). Overall, these data not only confirmed the importance of stabilin receptors in the recognition and uptake of srLNPs within mammalian LSECs but, importantly, highlighted the translational and predictive potential of the embryonic zebrafish as an early stage in vivo screening platform for new LNP designs.

## 3. Discussion

Based on a comprehensive understanding of the dominant nano–biointeractions involved,^[38]^ here, we have rationally designed a LNP–mRNA platform capable of preferentially targeting the hepatic RES, leading to enhanced mRNA expression within hepatic RES cell types. This biocentric approach to LNP design starkly contrasts with conventional empirical screening methods.^[17,19,20,88]^ Perhaps most importantly, this approach permits well-reasoned predictions of LNP in vivo fate without the need to screen endless variants in unnecessary animals. Given the potential chemical space of a LNP is virtually limitless, biocentric and informed design criteria will be essential in expediting the discovery of new LNP designs with enhanced functionality and efficacy.

In the case of preferential hepatic RES targeting, we have previously shown that stabilin-mediated recognition and uptake within SECs predominate over a wide range of anionic nanoparticle chemistries, both natural and synthetic.^[38]^ This generality gives us high confidence that alternative, anionic LNP–RNA technologies (with a measured surface charge of <−15 mV and optimally between 20 and 100 nm in size) will also reroute to the hepatic RES. These general guiding principles offer ample room for LNP optimization (e.g., retrofitting of new ionizable lipids^[89-91]^ and/or sterol components,^[52]^ chemically modified RNA^[92-94]^ and/or the use of miRNA suppression to improve target specificity^[95]^), and, as such, we firmly believe anionic LNP formulations should form the basis of future gene therapies against liver-specific and systemic diseases,^[28,96]^ including (auto)immune diseases,^[37]^ in which hepatic RES cell types play a central role.^[36]^

Comparing our srLNPs with existing (and empirically discovered) LNP technologies that have shown preferential RNA delivery to nonparenchymal hepatic cell types and/or nonhepatic cells, it is notable that none so far have possessed a measured surface charge of <−15 mV (where reported). These technologies are therefore unlikely to exploit a charge-dependent, stabilin-mediated pathway of LNP recognition and uptake. Preferential delivery of mRNA to liver ECs has, for example, been achieved through the replacement of cholesterol with either cholesteryl oleate or oxidized cholesterol components.^[20,21]^ It is possible that these systems conform to a charge- and/or stabilin-mediated mechanism of uptake within LSECs, as has been observed for both oxidized LDL (OxLDL) and acetylated LDL (AcLDL).^[29,97]^ However, in the absence of reported zeta potentials, and given both sterol reagents are charge neutral and likely predominate within the LNP core, LNPs containing cholesteryl oleate or oxidized cholesterol components are more likely exploiting an alternative, charge-independent mechanism of LNP recognition and uptake within liver ECs. Alternatively, exclusive LNP-mediated RNA delivery to the spleen has been achieved by adding the anionic phospholipid, 18PA (optimally 40 mol%), to the lipid composition of Onpattro.^[17]^ Given the near-neutral surface charge (−5.57 mV) of this formulation and the evident lack of liver targeting (where stabilins are highly expressed), however, this splenic tropism is highly unlikely to be either charge- or stabilin-dependent.

These studies do highlight, however, the complex interplay between LNP compositional makeup, biophysical properties, LNP ultrastructure, and in vivo LNP fate. To this end, comprehensive chemical and biological characterization of new LNP designs is an absolute necessity. Unfortunately, in vivo assessment of LNP fate is generally limited by the practicalities and costs of large-scale studies in conventional animal models (e.g., mice and rats). To this end, our results highlight that the embryonic zebrafish can be a powerful addition to LNP discovery pipelines.^[39]^ As a screening and optimization tool, zebrafish embryos enable real-time, in vivo visualization of total LNP injected doses at cellular resolution. Furthermore, with a conserved repertoire of RES and liver cell types, as well as soluble lipid transport proteins and receptors, the data acquired within these animals can provide qualitative predictions of cell-specific LNP recognition and uptake within key mammalian RES organs. As a fundamental tool to elucidate biological mechanisms underpinning LNP transport and RNA delivery, the short generational time of the zebrafish (≈3 months), the extensive repertoire of established (fluorescent) transgenic lines and antibodies,^[98,99]^ optimized techniques for genetic manipulation (e.g., clustered regularly interspaced short palindromic repeats/CRISPR-associated protein 9 (CRISPR/Cas),^[100]^ and advanced imaging techniques enable key nano–biointeractions underpinning LNP fate in vivo to be rapidly assessed and confirmed. In this case, the zebrafish embryo offered a unique opportunity to assess the combined role of stabilin-1 and −2 using established double KO mutant embryos. Analogous experiments in mice are severely complicated by the premature mortality and severe glomerular fibrosis of *stab1*^*−/−*^/*stab2*^*−/−*^ DKO mouse models.^[34]^

## 4. Conclusion

The widespread evaluation of LNP-based mRNA therapies as prophylactic vaccines,^[101,102]^ notably against COVID-19,^[103-106]^ has provided further proof of the broad therapeutic potential of these platform mRNA technologies. Despite the obvious differences in therapeutic target, mode of action, and injection site, however, all LNP–mRNA vaccine candidates, to date, closely resemble the lipid composition of Onpattro. In particular, LNP surface lipids (i.e., “helper” phospholipids and PEG lipids), cholesterol content, and overall lipid composition are strikingly similar between different clinical formulations. In line with our observations of LNPs with Onpattro-like surfaces, these vaccines elicit broad, nonspecific mRNA expression profiles across a wide range of cell types, both at the site of injection and within the liver.^[103]^ However, while the ability to leverage a wide array of cell types to produce a therapeutic protein may be safe and effective as a systemic secreted therapy (i.e., suitable for vaccine application), the lack of LNP designs capable of preferential RNA delivery to specific (diseased) cells and tissues in the body remains a major limitation of these “plug-and-play” technologies. Overall, this work highlights that a biocentric approach to LNP discovery, based on robust prior knowledge of the dominant nano–biointeractions involved, is a logical and highly effective approach to propel the discovery of new and enhanced LNP designs and can accelerate the widespread clinical application of these game changing technologies.

## Supplementary Material

supporting information

## Data Availability

The data that support the findings of this study are available from the corresponding author upon reasonable request.

## References

[1] Nat. Med 2019, 25, 1321. 31501598 10.1038/s41591-019-0580-6

[2] Dammes N, Peer D, Trends Pharmacol. Sci 2020, 41, 755. 32893005 10.1016/j.tips.2020.08.004 PMC7470715

[3] Yin H, Kanasty RL, Eltoukhy AA, Vegas AJ, Dorkin JR, Anderson DG, Nat. Rev. Genet 2014, 15, 541. 25022906 10.1038/nrg3763

[4] Cullis PR, Hope MJ, Mol. Ther 2017, 25, 1467. 28412170 10.1016/j.ymthe.2017.03.013 PMC5498813

[5] Kulkarni JA, Witzigmann D, Chen S, Cullis PR, van der Meel R, Acc. Chem. Res 2019, 52, 2435. 31397996 10.1021/acs.accounts.9b00368

[6] Akinc A, Maier MA, Manoharan M, Fitzgerald K, Jayaraman M, Barros S, Ansell S, Du X, Hope MJ, Madden TD, Mui BL, Semple SC, Tam YK, Ciufolini M, Witzigmann D, Kulkarni JA, van der Meel R, Cullis PR, Nat. Nanotechnol 2019, 14, 1084. 31802031 10.1038/s41565-019-0591-y

[7] Adams D, Gonzalez-Duarte A, O’Riordan WD, Yang CC, Ueda M, Kristen AV, Tournev I, Schmidt HH, Coelho T, Berk JL, Lin KP, Vita G, Attarian S, Planté-Bordeneuve V, Mezei MM, Campistol JM, Buades J, Brannagan TH, Kim BJ, Oh J, Parman Y, Sekijima Y, Hawkins PN, Solomon SD, Polydefkis M, Dyck PJ, Gandhi PJ, Goyal S, Chen J, Strahs AL, , N. Engl. J. Med 2018, 379, 11. 29972753 10.1056/NEJMoa1716153

[8] Kumar V, Qin J, Jiang Y, Duncan RG, Brigham B, Fishman S, Nair JK, Akinc A, Barros SA, Kasperkovitz PV, Mol. Ther.–Nucleic Acids 2014, 3, e210. 25405467 10.1038/mtna.2014.61 PMC4459547

[9] Mui BL, Tam YK, Jayaraman M, Ansell SM, Du X, Tam YYC, Lin PJ, Chen S, Narayanannair JK, Rajeev KG, Manoharan M, Akinc A, Maier MA, Cullis P, Madden TD, Hope MJ, Mol. Ther.–Nucleic Acids 2013, 2, e139. 24345865 10.1038/mtna.2013.66 PMC3894582

[10] Akinc A, Querbes W, De S, Qin J, Frank-Kamenetsky M, Jayaprakash KN, Jayaraman M, Rajeev KG, Cantley WL, Dorkin JR, Butler JS, Qin L, Racie T, Sprague A, Fava E, Zeigerer A, Hope MJ, Zerial M, Sah DWY, Fitzgerald K, Tracy MA, Manoharan M, Koteliansky V, de Fougerolles A, Maier MA, Mol. Ther 2010, 18, 1357. 20461061 10.1038/mt.2010.85 PMC2911264

[11] Nguyen J, Szoka FC, Acc. Chem. Res 2012, 45, 1153. 22428908 10.1021/ar3000162 PMC3399092

[12] Sato Y, Matsui H, Yamamoto N, Sato R, Munakata T, Kohara M, Harashima H, J. Controlled Release 2017, 266, 216. 10.1016/j.jconrel.2017.09.044 28986168

[13] DeRosa F, Guild B, Karve S, Smith L, Love K, Dorkin JR, Kauffman KJ, Zhang J, Yahalom B, Anderson DG, Heartlein MW, Gene Ther. 2016, 23, 699. 27356951 10.1038/gt.2016.46 PMC5059749

[14] Ramaswamy S, Tonnu N, Tachikawa K, Limphong P, Vega JB, Karmali PP, Chivukula P, Verma IM, Proc. Natl. Acad. Sci. USA 2017, 114, E1941. 28202722 10.1073/pnas.1619653114 PMC5347596

[15] Chen S, Tam YYC, Lin PJC, Leung AKK, Tam YK, Cullis PR, J. Controlled Release 2014, 196, 106. 10.1016/j.jconrel.2014.09.025 25285610

[16] Sago CD, Lokugamage MP, Islam FZ, Krupczak BR, Sato M, Dahlman JE, J. Am. Chem. Soc 2018, 140, 17095. 30394729 10.1021/jacs.8b08976 PMC6556374

[17] Cheng Q, Wei T, Farbiak L, Johnson LT, Dilliard SA, Siegwart DJ, Nat. Nanotechnol 2020, 15, 313. 32251383 10.1038/s41565-020-0669-6 PMC7735425

[18] Lokugamage MP, Sago CD, Gan Z, Krupczak BR, Dahlman JE, Adv. Mater 2019, 31, 1902251. 10.1002/adma.201902251 PMC6819129 31465135

[19] Kedmi R, Veiga N, Ramishetti S, Goldsmith M, Rosenblum D, Dammes N, Hazan-Halevy I, Nahary L, Leviatan-Ben-Arye S, Harlev M, Behlke M, Benhar I, Lieberman J, Peer D, Nat. Nanotechnol 2018, 13, 214. 29379205 10.1038/s41565-017-0043-5

[20] Paunovska K, Gil CJ, Lokugamage MP, Sago CD, Sato M, Lando GN, Gamboa Castro M, Bryksin AV, Dahlman JE, ACS Nano 2018, 12, 8341. 30016076 10.1021/acsnano.8b03640 PMC6115295

[21] Paunovska K, Da Silva Sanchez AJ, Sago CD, Gan Z, Lokugamage MP, Islam FZ, Kalathoor S, Krupczak BR, Dahlman JE, Adv. Mater 2019, 31, 1807748. 10.1002/adma.201807748 PMC6445717 30748040

[22] Witzigmann D, Hak S, van der Meel R, J. Controlled Release 2018, 290, 138. 10.1016/j.jconrel.2018.10.011 30308257

[23] Trefts E, Gannon M, Wasserman DH, Curr. Biol 2017, 27, R1147. 29112863 10.1016/j.cub.2017.09.019 PMC5897118

[24] Wisse E, J. Ultrastruct. Res 1970, 31, 125. 5442603 10.1016/s0022-5320(70)90150-4

[25] Braet F, Wisse E, Comp. Hepatol 2002, 1, 1. 12437787 10.1186/1476-5926-1-1 PMC131011

[26] Smedsrød B, Pertoft H, Gustafson S, Laurent TC, Biochem. J 1990, 266, 313 2156492 10.1042/bj2660313 PMC1131134

[27] Sørensen KK, Simon-Santamaria J, McCuskey RS, Smedsrød B, Compr. Physiol 2015, 5, 1751. 26426467 10.1002/cphy.c140078

[28] Poisson J, Lemoinne S, Boulanger C, Durand F, Moreau R, Valla D, Rautou P-E, J. Hepatol 2017, 66, 212. 27423426 10.1016/j.jhep.2016.07.009

[29] Li R, Oteiza A, Sørensen KK, McCourt P, Olsen R, Smedsrød B, Svistounov D, Am. J. Physiol. Gastrointest. Liver Physiol 2010, 300, G71. 21030611 10.1152/ajpgi.00215.2010 PMC3025507

[30] Smedsrød B, Comp. Hepatol 2004, 3, S22. 14960174 10.1186/1476-5926-2-S1-S22 PMC2409441

[31] Ganesan LP, Mohanty S, Kim J, Clark KR, Robinson JM, Anderson CL, PLoS Pathog. 2011, 7, e1002281. 21980295 10.1371/journal.ppat.1002281 PMC3182912

[32] Mates JM, Yao Z, Cheplowitz AM, Suer O, Phillips GS, Kwiek JJ, Rajaram MVS, Kim J, Robinson JM, Ganesan LP, Anderson CL, Front. Immunol 2017, 8, 35. 28167948 10.3389/fimmu.2017.00035 PMC5256111

[33] Sørensen KK, McCourt P, Berg T, Crossley C, Le Couteur D, Wake K, Smedsrød B, Am. J. Physiol.: Regul., Integr. Comp. Physiol 2012, 303, R1217. 23076875 10.1152/ajpregu.00686.2011

[34] Schledzewski K, Géraud C, Arnold B, Wang S, Gröne H-J, Kempf T, Wollert KC, Straub BK, Schirmacher P, Demory A, Schönhaber H, Gratchev A, Dietz L, Thierse H-J, Kzhyshkowska J, Goerdt S, J. Clin. Invest 2011, 121, 703. 21293057 10.1172/JCI44740 PMC3026735

[35] McCourt PAG, Hansen B, Svistuonov D, Johansson S, Longati P, Schledzewski K, Kzhyshkowska J, Goerdt S, Johansson S, Smedsrød B, Comp. Hepatol 2004, 3, S24. 14960176 10.1186/1476-5926-2-S1-S24 PMC2410243

[36] Wilkinson AL, Qurashi M, Shetty S, Front. Physiol 2020, 11, 990. 32982772 10.3389/fphys.2020.00990 PMC7485256

[37] Shetty S, Lalor PF, Adams DH, Nat. Rev. Gastroenterol. Hepatol 2018, 15, 555. 29844586 10.1038/s41575-018-0020-y PMC7096836

[38] Campbell F, Bos FL, Sieber S, Arias-Alpizar G, Koch BE, Huwyler J, Kros A, Bussmann J, ACS Nano 2018, 12, 2138. 29320626 10.1021/acsnano.7b06995 PMC5876619

[39] Sieber S, Grossen P, Bussmann J, Campbell F, Kros A, Witzigmann D, Huwyler J, Adv. Drug Delivery Rev 2019, 151-152, 152. 10.1016/j.addr.2019.01.001 30615917

[40] Seternes T, Sørensen K, Smedsrød B, Proc. Natl. Acad. Sci. USA 2002, 99, 7594. 12032328 10.1073/pnas.102173299 PMC124295

[41] Bhandari S, Larsen AK, McCourt P, Smedsrød B, Sørensen KK, Front. Physiol 2021, 12, 757469. 34707514 10.3389/fphys.2021.757469 PMC8542980

[42] Hayashi Y, Takamiya M, Jensen PB, Ojea-Jiménez I, Claude H, Antony C, Kjaer-Sorensen K, Grabher C, Boesen T, Gilliland D, Oxvig C, Strähle U, Weiss C, ACS Nano 2020, 14, 1665. 31922724 10.1021/acsnano.9b07233

[43] Evers MJW, Kulkarni JA, van der Meel R, Cullis PR, Vader P, Schiffelers RM, Small Methods 2018, 2, 1700375.

[44] Arteta MY, Kjellman T, Bartesaghi S, Wallin S, Wu X, Kvist AJ, Dabkowska A, Székely N, Radulescu A, Bergenholtz J, Lindfors L, Proc. Natl. Acad. Sci. USA 2018, 115, E3351. 29588418 10.1073/pnas.1720542115 PMC5899464

[45] Yamamoto T, Ryan RO, Biochem. Biophys. Res. Commun 2007, 354, 820. 17258176 10.1016/j.bbrc.2007.01.066

[46] Kulkarni JA, Witzigmann D, Leung J, Tam YYC, Cullis PR, Nanoscale 2019, 11, 21733. 31713568 10.1039/c9nr09347h

[47] Kulkarni JA, Darjuan MM, Mercer JE, Chen S, van der Meel R, Thewalt JL, Tam YYC, Cullis PR, ACS Nano 2018, 12, 4787. 29614232 10.1021/acsnano.8b01516

[48] Leung AKK, Hafez IM, Baoukina S, Belliveau NM, Zhigaltsev IV, Afshinmanesh E, Tieleman DP, Hansen CL, Hope MJ, Cullis PR, J. Phys. Chem. C 2012, 116, 18440. 10.1021/jp303267y PMC3434764 22962627

[49] Kulkarni JA, Witzigmann D, Leung J, van der Meel R, Zaifman J, Darjuan MM, Grisch-Chan HM, Thöny B, Tam YYC, Cullis PR, Nanoscale 2019, 11, 9023. 31021343 10.1039/c9nr02004g

[50] Crawford R, Dogdas B, Keough E, Haas RM, Wepukhulu W, Krotzer S, Burke PA, Sepp-Lorenzino L, Bagchi A, Howell BJ, Int. J. Pharm 2011, 403, 237. 20974237 10.1016/j.ijpharm.2010.10.025

[51] Eygeris Y, Patel S, Jozic A, Sahay G, Nano Lett. 2020, 20, 4543. 32375002 10.1021/acs.nanolett.0c01386

[52] Patel S, Ashwanikumar N, Robinson E, Xia Y, Mihai C, Griffith JP, Hou S, Esposito AA, Ketova T, Welsher K, Joyal JL, Almarsson Ö, Sahay G, Nat. Commun 2020, 11, 983. 32080183 10.1038/s41467-020-14527-2 PMC7033178

[53] Aderem A, Underhill DM, Annu. Rev. Immunol 1999, 17, 593. 10358769 10.1146/annurev.immunol.17.1.593

[54] Paul D, Achouri S, Yoon Y-Z, Herre J, Bryant CE, Cicuta P, Biophys. J 2013, 105, 1143. 24010657 10.1016/j.bpj.2013.07.036 PMC3762343

[55] Zapotoczny B, Szafranska K, Owczarczyk K, Kus E, Chlopicki S, Szymonski M, Sci. Rep 2017, 7, 7994. 28801568 10.1038/s41598-017-08555-0 PMC5554186

[56] Arias-Alpizar G, Koch B, Hamelmann NM, Neustrup MA, Paulusse JMJ, Jiskoot W, Kros A, Bussmann J, Nanomedicine 2021, 34, 102395. 33838334 10.1016/j.nano.2021.102395

[57] Reiser A, Woschée D, Mehrotra N, Krzysztón R, Strey HH, Rädler JO, Integr. Biol 2019, 11, 362. 10.1093/intbio/zyz030 31850498

[58] Balleza E, Kim JM, Cluzel P, Nat. Methods 2018, 15, 47. 29320486 10.1038/nmeth.4509 PMC5765880

[59] Lopes S, Yang X, Müller J, Carney T, Mcadow A, Rauch G-J, Jacoby A, Hurst L, Delfino-Machin M, Haffter P, Geisler R, Johnson S, Ward A, Kelsh R, PLoS Genet. 2008, 4, e1000026. 18369445 10.1371/journal.pgen.1000026 PMC2265441

[60] Leonhardt C, Schwake G, Stögbauer TR, Rappl S, Kuhr J-T, Ligon TS, Rädler JO, Nanomedicine 2014, 10, 679. 24333584 10.1016/j.nano.2013.11.008

[61] Patel S, Kim J, Herrera M, Mukherjee A, Kabanov AV, Sahay G, Adv. Drug Delivery Rev 2019, 144, 90. 10.1016/j.addr.2019.08.004 PMC6986687 31419450

[62] Gilleron J, Querbes W, Zeigerer A, Borodovsky A, Marsico G, Schubert U, Manygoats K, Seifert S, Andree C, Stöter M, Epstein-Barash H, Zhang L, Koteliansky V, Fitzgerald K, Fava E, Bickle M, Kalaidzidis Y, Akinc A, Maier M, Zerial M, Nat. Biotechnol 2013, 31, 638. 23792630 10.1038/nbt.2612

[63] Sahay G, Querbes W, Alabi C, Eltoukhy A, Sarkar S, Zurenko C, Karagiannis E, Love K, Chen D, Zoncu R, Buganim Y, Schroeder A, Langer R, Anderson DG, Nat. Biotechnol 2013, 31, 653. 23792629 10.1038/nbt.2614 PMC3814166

[64] Miller CM, Donner AJ, Blank EE, Egger AW, Kellar BM, Østergaard ME, Seth PP, Harris EN, Nucleic Acids Res. 2016, 44, 2782. 26908652 10.1093/nar/gkw112 PMC4824115

[65] Wilkins BJ, Pack M, Compr. Physiol 2013, 3, 1213. 23897685 10.1002/cphy.c120021 PMC4784975

[66] Wang S, Miller SR, Ober EA, Sadler KC, Curr. Top. Dev. Biol 2017, 124, 161. 28335859 10.1016/bs.ctdb.2016.11.012 PMC6450094

[67] Korzh S, Pan X, Garcia-Lecea M, Winata CL, Pan X, Wohland T, Korzh V, Gong Z, BMC Dev. Biol 2008, 8, 84. 18796162 10.1186/1471-213X-8-84 PMC2564926

[68] Mudumana SP, Wan H, Singh M, Korzh V, Gong Z, Dev. Dyn 2004, 230, 165. 15108321 10.1002/dvdy.20032

[69] Her GM, Chiang C-C, Chen W-Y, Wu J-L, FEBS Lett. 2003, 538, 125. 12633865 10.1016/s0014-5793(03)00157-1

[70] Yin C, Evason KJ, Maher JJ, Stainier DYR, Hepatology 2012, 56, 1958. 22488653 10.1002/hep.25757 PMC3407311

[71] Cheng D, Morsch M, Shami GJ, Chung RS, Braet F, Exp. Cell Res 2019, 374, 162. 30496757 10.1016/j.yexcr.2018.11.020

[72] Otis JP, Zeituni EM, Thierer JH, Anderson JL, Brown AC, Boehm ED, Cerchione DM, Ceasrine AM, Avraham-Davidi I, Tempelhof H, Yaniv K, Farber SA, Dis. Models Mech 2015, 8, 295. 10.1242/dmm.018754 PMC4348566 25633982

[73] Babin PJ, Thisse C, Durliat M, Andre M, Akimenko M-A, Thisse B, Proc. Natl. Acad. Sci. USA 1997, 94, 8622. 9238027 10.1073/pnas.94.16.8622 PMC23048

[74] Liu C, Kim YS, Kim J, Pattison J, Kamaid A, Miller YI, J. Lipid Res 2018, 59, 391. 29187523 10.1194/jlr.D081521 PMC5794413

[75] O’Hare EA, Wang X, Montasser ME, Chang Y-PC, Mitchell BD, Zaghloul NA, J. Lipid Res 2014, 55, 2242. 25201834 10.1194/jlr.M046540 PMC4617127

[76] Huang Y, Mahley RW, Neurobiol. Dis 2014, 72 Pt A, 3. 25173806 10.1016/j.nbd.2014.08.025 PMC4253862

[77] Mahley RW, Weisgraber KH, Huang Y, J. Lipid Res 2009, 50, S183. 19106071 10.1194/jlr.R800069-JLR200 PMC2674716

[78] Schlegel A, Front. Endocrinol 2016, 7, 159. 10.3389/fendo.2016.00159 PMC5159437 28018294

[79] Wang D, El-Amouri SS, Dai M, Kuan C-Y, Hui DY, Brady RO, Pan D, Proc. Natl. Acad. Sci. USA 2013, 110, 2999. 23382178 10.1073/pnas.1222742110 PMC3581871

[80] Jiang Y, Zhang J, Meng F, Zhong Z, ACS Nano 2018, 12, 11070. 30395440 10.1021/acsnano.8b05265

[81] Böckenhoff A, Cramer S, Wölte P, Knieling S, Wohlenberg C, Gieselmann V, Galla H-J, Matzner U, J. Neurosci 2014, 34, 3122. 24573272 10.1523/JNEUROSCI.4785-13.2014 PMC6795304

[82] Shi B, Keough E, Matter A, Leander K, Young S, Carlini E, Sachs AB, Tao W, Abrams M, Howell B, Sepp-Lorenzino L, J. Histochem. Cytochem 2011, 59, 727. 21804077 10.1369/0022155411410885 PMC3261601

[83] Mayer LD, Cullis PR, Bally MB, in Medical Applications of Liposomes (Eds: Lasic DD, Papahadjopoulos D), Elsevier Science B.V., Amsterdam, The Netherlands 1998, pp. 231–257.

[84] Sago CD, Krupczak BR, Lokugamage MP, Gan Z, Dahlman JE, Cell. Mol. Bioeng 2019, 12, 389. 31719922 10.1007/s12195-019-00573-4 PMC6816632

[85] “Moderna Announces Positive Phase 1 Results for the First Systemic Messenger RNA Therapeutic Encoding a Secreted Protein (mRNA-1944),” https://investors.modernatx.com/news-releases/news-release-details/moderna-announces-positive-phase-1-results-first-systemic (accessed: December 2019).

[86] Schulze RJ, Schott MB, Casey CA, Tuma PL, McNiven MA, J. Cell Biol 2019, 218, 2096. 31201265 10.1083/jcb.201903090 PMC6605791

[87] Hirose Y, Saijou E, Sugano Y, Takeshita F, Nishimura S, Nonaka H, Chen Y-R, Sekine K, Kido T, Nakamura T, Kato S, Kanke T, Nakamura K, Nagai R, Ochiya T, Miyajima A, Proc. Natl. Acad. Sci. USA 2012, 109, 4263. 22371575 10.1073/pnas.1117560109 PMC3306694

[88] Paunovska K, Sago CD, Monaco CM, Hudson WH, Castro MG, Rudoltz TG, Kalathoor S, Vanover DA, Santangelo PJ, Ahmed R, Bryksin AV, Dahlman JE, Nano Lett. 2018, 18, 2148. 29489381 10.1021/acs.nanolett.8b00432 PMC6054134

[89] Rietwyk S, Peer D, ACS Nano 2017, 11, 7572. 28727419 10.1021/acsnano.7b04734

[90] Miao L, Li L, Huang Y, Delcassian D, Chahal J, Han J, Shi Y, Sadtler K, Gao W, Lin J, Doloff JC, Langer R, Anderson DG, Nat. Biotechnol 2019, 37, 1174. 31570898 10.1038/s41587-019-0247-3

[91] Hassett KJ, Benenato KE, Jacquinet E, Lee A, Woods A, Yuzhakov O, Himansu S, Deterling J, Geilich BM, Ketova T, Mihai C, Lynn A, McFadyen I, Moore MJ, Senn JJ, Stanton MG, Almarsson Ö, Ciaramella G, Brito LA, Mol. Ther.–Nucleic Acids 2019, 15, 1. 30785039 10.1016/j.omtn.2019.01.013 PMC6383180

[92] Khvorova A, Watts JK, Nat. Biotechnol 2017, 35, 238. 28244990 10.1038/nbt.3765 PMC5517098

[93] Ku SH, Jo SD, Lee YK, Kim K, Kim SH, Adv. Drug Delivery Rev 2016, 104, 16. 10.1016/j.addr.2015.10.015 26549145

[94] Shen X, Corey DR, Nucleic Acids Res. 2018, 46, 1584. 29240946 10.1093/nar/gkx1239 PMC5829639

[95] Jain R, Frederick JP, Huang EY, Burke KE, Mauger DM, Andrianova EA, Farlow SJ, Siddiqui S, Pimentel J, Cheung-Ong K, McKinney KM, Köhrer C, Moore MJ, Chakraborty T, Nucleic Acid Ther. 2018, 28, 285. 30088967 10.1089/nat.2018.0734 PMC6157376

[96] Ni Y, Li J-M, Liu M-K, Zhang T-T, Wang D-P, Zhou W-H, Hu L-Z, Lv W-L, World J. Gastroenterol 2017, 23, 7666. 29209108 10.3748/wjg.v23.i43.7666 PMC5703927

[97] De Rijke YB, Biessen EA, Vogelezang CJ, van Berkel TJ, Biochem. J 1994, 304, 69. 7998959 10.1042/bj3040069 PMC1137453

[98] Staudt N, Müller-Sienerth N, Fane-Dremucheva A, Yusaf SP, Millrine D, Wright GJ, Biochem. Biophys. Res. Commun 2015, 456, 527. 25490391 10.1016/j.bbrc.2014.11.123 PMC4297863

[99] Burket CT, Montgomery JE, Thummel R, Kassen SC, LaFave MC, Langenau DM, Zon LI, Hyde DR, Transgenic Res. 2008, 17, 265. 17968670 10.1007/s11248-007-9152-5 PMC3660017

[100] Varshney GK, Pei W, LaFave MC, Idol J, Xu L, Gallardo V, Carrington B, Bishop K, Jones M, Li M, Harper U, Huang SC, Prakash A, Chen W, Sood R, Ledin J, Burgess SM, Genome Res. 2015, 25, 1030. 26048245 10.1101/gr.186379.114 PMC4484386

[101] Pardi N, Hogan MJ, Porter FW, Weissman D, Nat. Rev. Drug Discovery 2018, 17, 261. 29326426 10.1038/nrd.2017.243 PMC5906799

[102] John S, Yuzhakov O, Woods A, Deterling J, Hassett K, Shaw CA, Ciaramella G, Vaccine 2018, 36, 1689. 29456015 10.1016/j.vaccine.2018.01.029

[103] Zhang N-N, Li X-F, Deng Y-Q, Zhao H, Huang Y-J, Yang G, Huang W-J, Gao P, Zhou C, Zhang R-R, Guo Y, Sun S-H, Fan H, Zu S-L, Chen Q, He Q, Cao T-S, Huang X-Y, Qiu H-Y, Nie J-H, Jiang Y, Yan H-Y, Ye Q, Zhong X, Xue X-L, Zha Z-Y, Zhou D, Yang X, Wang Y-C, Ying B, , Cell 2020, 182, 1271. 32795413 10.1016/j.cell.2020.07.024 PMC7377714

[104] Jackson LA, Anderson EJ, Rouphael NG, Roberts PC, Makhene M, Coler RN, McCullough MP, Chappell JD, Denison MR, Stevens LJ, Pruijssers AJ, McDermott A, Flach B, Doria-Rose NA, Corbett KS, Morabito KM, O’Dell S, Schmidt SD, Swanson PA, Padilla M, Mascola JR, Neuzil KM, Bennett H, Sun W, Peters E, Makowski M, Albert J, Cross K, Buchanan W, Pikaart-Tautges R, , N. Engl. J. Med 2020, 383, 1920. 32663912 10.1056/NEJMoa2022483 PMC7377258

[105] McKay PF, Hu K, Blakney AK, Samnuan K, Brown JC, Penn R, Zhou J, Bouton CR, Rogers P, Polra K, Lin PJC, Barbosa C, Tam YK, Barclay WS, Shattock RJ, Nat. Commun 2020, 11, 3523. 32647131 10.1038/s41467-020-17409-9 PMC7347890

[106] Lu J, Lu G, Tan S, Xia J, Xiong H, Yu X, Qi Q, Yu X, Li L, Yu H, Xia N, Zhang T, Xu Y, Lin J, Cell Res. 2020, 30, 936. 32801356 10.1038/s41422-020-00392-7 PMC7429369

