# Supplementary material for: Anionic Lipid Nanoparticles Preferentially Deliver mRNA to the Hepatic Reticuloendothelial System
Source: Adv Mater. Author manuscript; Available in PMC 2022 Oct 1. (PMC9461706; doi:10.1002/adma.202201095)
Supplement: supporting information [file NIHMS1833154-supplement-supporting_information.pdf]

ADVANCED MATERIALS

Supporting Information

for *Adv. Mater.,* DOI: 10.1002/adma.202201095

Anionic Lipid Nanoparticles Preferentially Deliver mRNA to the Hepatic Reticuloendothelial System

Roy Pattipeiluhu, Gabriela Arias-Alpizar, Genc Basha, Karen Y. T. Chan, Jeroen Bussmann, Thomas H. Sharp, Mohammad-Amin Moradi, Nico Sommerdijk, Edward N. Harris, Pieter R. Cullis, Alexander Kros, Dominik Witzigmann, and Frederick Campbell**

Supporting Information for

Anionic Lipid Nanoparticles Preferentially Deliver mRNA to the Hepatic Reticuloendothelial System

Roy Pattipeiluhu, Gabriela Arias-Alpizar, Genc Basha, Karen Y. T. Chan, Jeroen Bussmann, Thomas H. Sharp, Mohammad-Amin Moradi, Nico Sommerdijk, Edward N. Harris, Pieter R. Cullis, Alexander Kros, Dominik Witzigmann and Frederick Campbell

Contents

Supporting Figures 3

Materials and Methods 15

Reagents 15

Synthesis of N-succinyl-3-amino-5-cholestene (3)[4] 16

Peptide synthesis 17

Chol-NH-ApoE_peptide synthesis and purification 17

Liposome Formulation 18

Lipid Nanoparticle (LNP) Formulation 18

Cryo-electron Microscopy Imaging and Quantification 19

Zebrafish Husbandry and Injections 20

Zebrafish confocal imaging acquisition and processing 21

Mouse husbandry, injection protocol and cell isolation 21

FACS analysis 23

Statistics and Reproducibility 23

Data Availability 24

Supporting Data 25

LC-MS spectrum of Chol-NH-ApoE_peptide 25

References 26

Supporting Figures

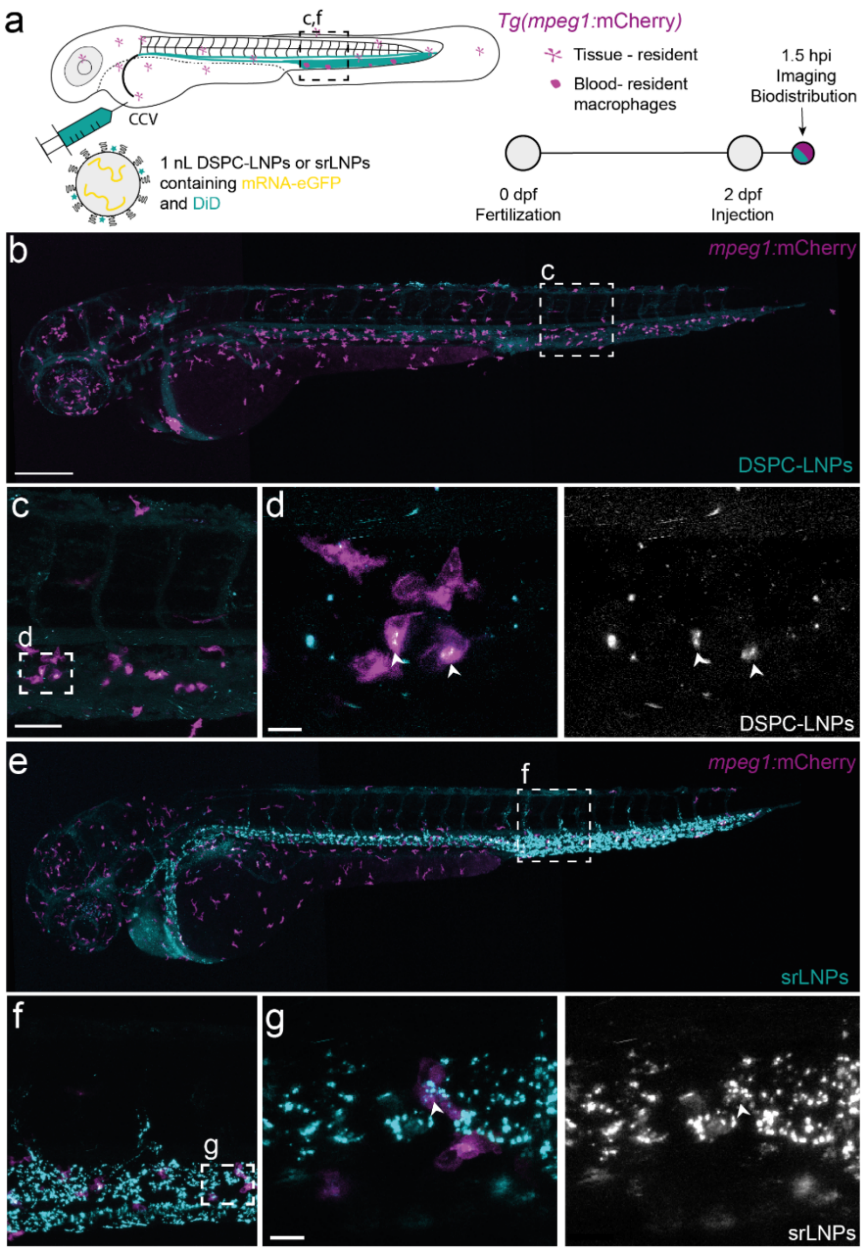

Figure S1. Biodistribution of DSPC-LNPs and srLNPs in *mpeg1:mCherry* transgenic zebrafish embryos at 1.5 hpi. Schematic showing the site of LNP injection *(i.v)* within the embryonic zebrafish (2 dpf) and imaging timeframe. LNPs contained DiD (cy5, 0.1 mol%) as fluorescent lipid probe and unlabeled eGFP mRNA. Transgenic *mpeg1:*mCherry zebrafish embryos stably express mCherry (magenta) within all macrophages. *Injected dose*: ~10 mM lipid, ~0.2 mg/kg mRNA. *Injection volume*: 1 nL. CCV – common cardinal vein (a) Whole embryo view (10x magnification), (b) tissue level view (40x magnification), and (c) a zoom of a maximum projection of three confocal z-stacks, showing fluorescent co-localization of DiD (LNP probe) and transgenic mCherry (white arrowheads), confirmed low-level DSPC-LNP uptake within blood resident macrophages. (d) Whole embryo view (10x magnification), (e) tissue level view (40x magnification) and (f) a zoom of a maximum projection of three confocal z-stacks, showing fluorescent co-localization of DiD (LNP probe) and transgenic mCherry (white arrowheads), confirmed simultaneous uptake of srLNPs within both SECs and blood resident macrophages. Scale bars: 200 µm (whole body), 50 µm (tissue level), 10 µm (zoom).

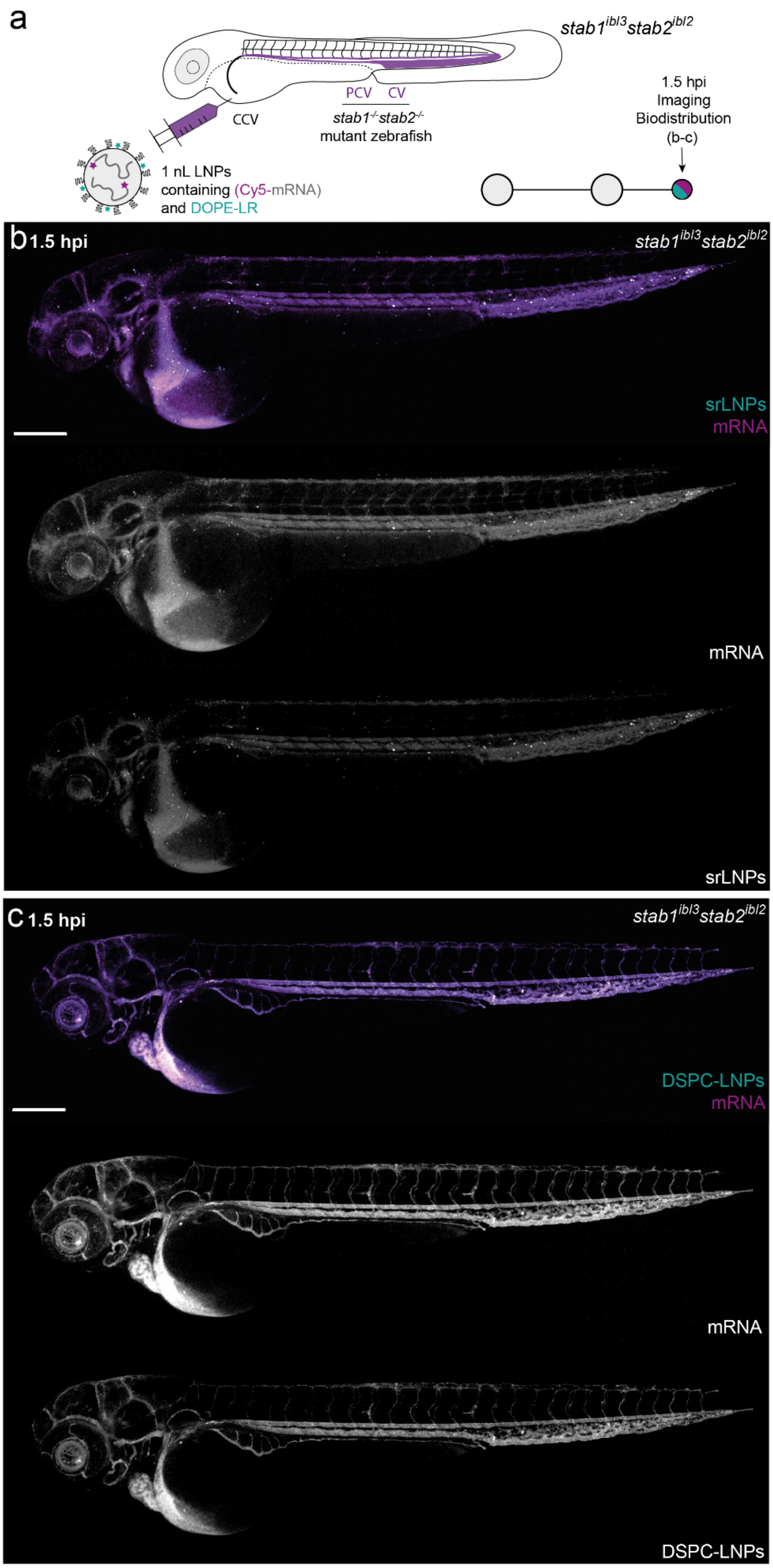

Figure S2. Biodistribution of srLNPs and DSPC-LNPs in double knock-out (*stab1*$^{-/-}$/*stab2*$^{-/-}$) mutant embryos at 1.5 hpi. (a) Schematic showing the site of LNP injection (*i.v.*) within double knockout (*stab1*^{ibl3}/*stab2*^{ibl2})[1] zebrafish embryos (2 dpf) and imaging timeframe. LNPs contained DOPE-LR (cyan, 0.2 mol%) as fluorescent lipid probe and Cy5-labelled eGFP mRNA (magenta) as fluorescent mRNA probe. *Injected dose*: ~10 mM lipid, ~0.2 mg/kg mRNA. *Injection volume*: ~1 nL. CV – cardinal vein; PCV – posterior

cardinal vein; CCV – common cardinal vein. (b,c) Whole embryo (10x magnification) views of srLNP and DSPC-LNP biodistribution within *stab1*$^{-/-}$/*stab2*$^{-/-}$ mutant embryos (2 dpf) at 1.5 hpi. In both cases, LNPs were mostly freely circulating throughout the vasculature of the embryo at 1.5 hpi. Scale bar: 200 µm.

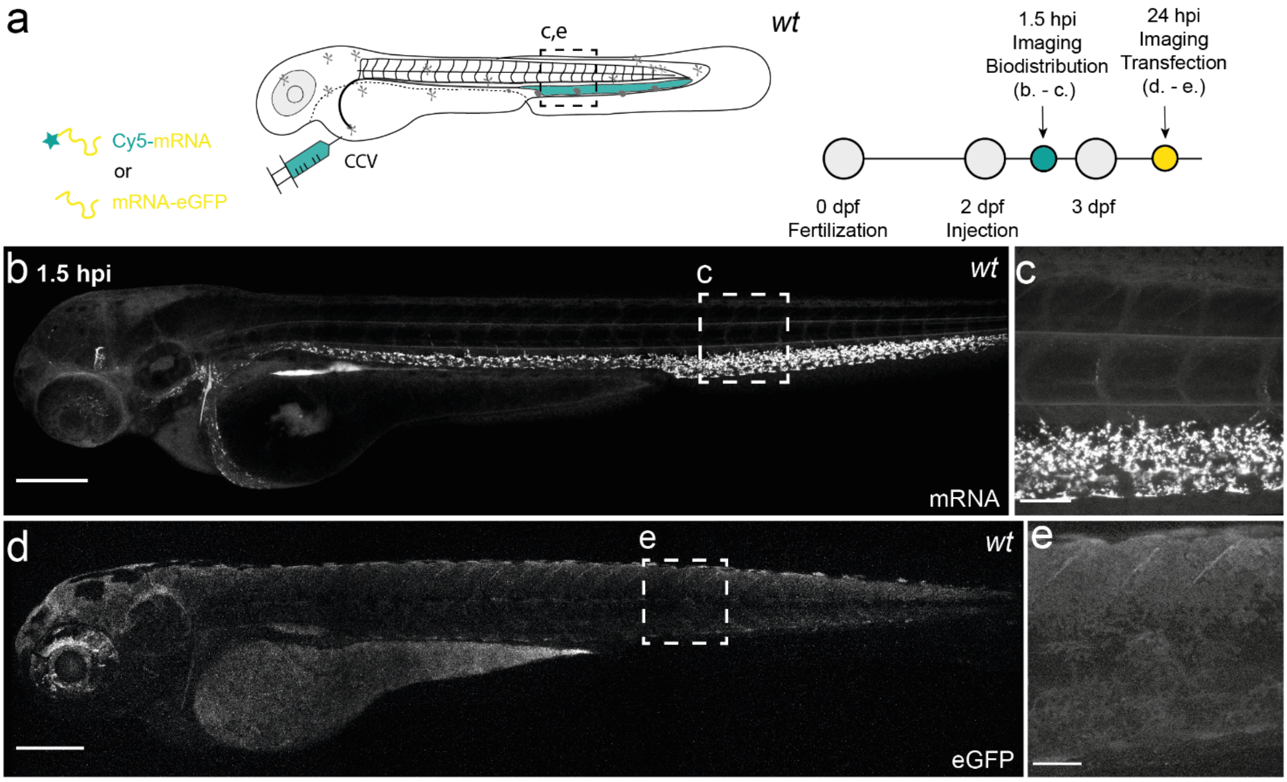

Figure S3. Biodistribution and expression of free eGFP mRNA in wildtype (AB/TL) embryonic zebrafish. (a) Schematic showing the site of free mRNA injection (*i.v.;* 0.2 mg/kg, 1 nL*)* within embryonic zebrafish (2 dpf) and imaging timeframe. CCV – common cardinal vein. (b,c) Whole embryo (10x magnification) and tissue level (40x magnification) views of free mRNA (Cy5-labelled) biodistribution at 1.5 hpi. Free mRNA primarily accumulated within SECs of the embryonic zebrafish at 1.5 hpi, likely mediated by Stabilin receptors.[2] Any phagocytotic uptake of free mRNA within blood resident macrophages cannot be clearly defined within the CHT of the wild-type embryo given the high fluorescence signal (Cy5) within overlapping SECs. (d,e) Whole embryo (10x magnification) and tissue level (40x magnification) views of eGFP expression (unlabeled mRNA) at 24 hpi. No significant eGFP expression is observed within SECs or blood resident macrophages of the embryonic fish. Scale bars: 200 µm (whole embryo) and 50 µm (tissue level).

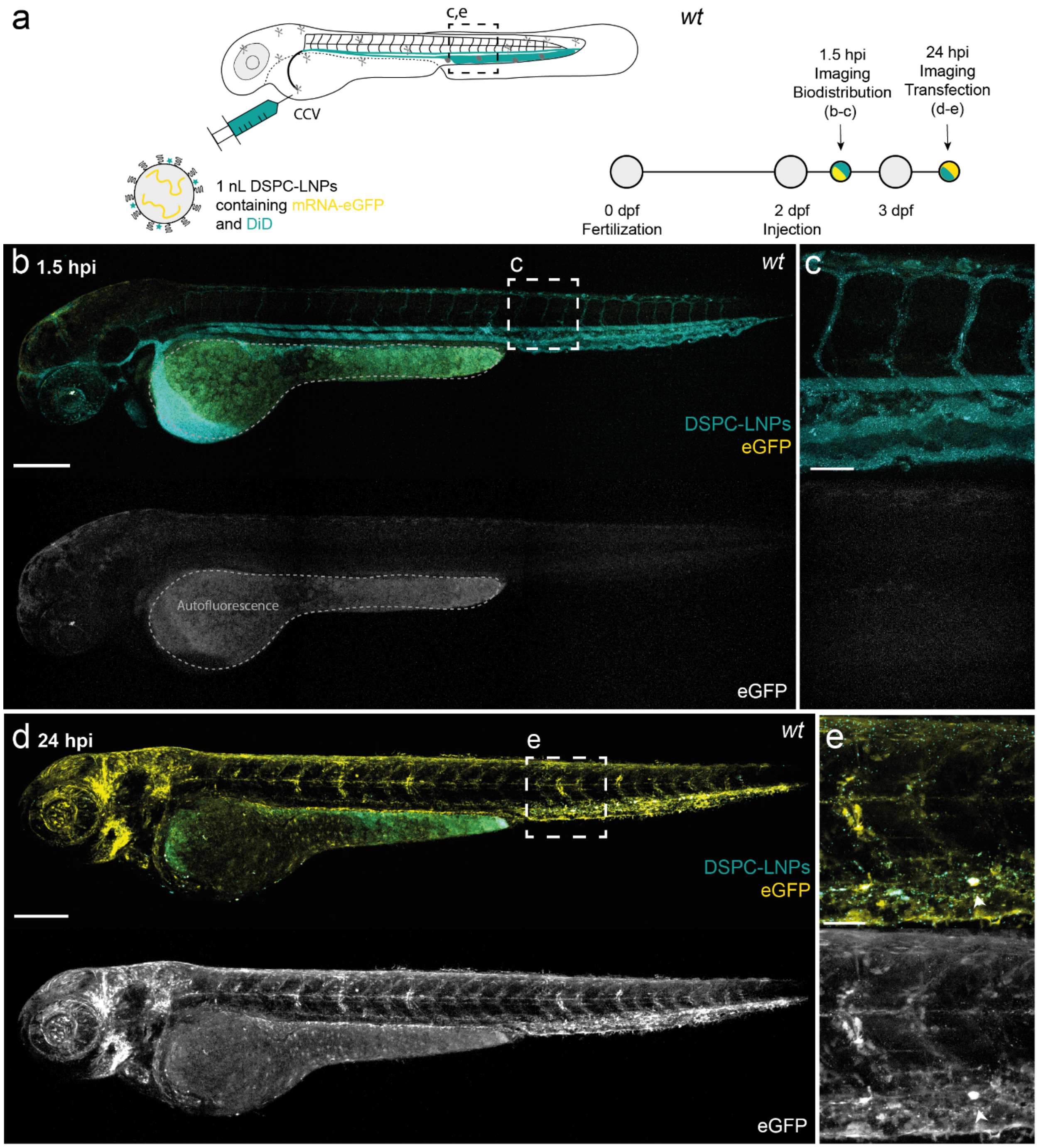

Figure S4. DSPC-LNP biodistribution and eGFP expression within wild-type (AB/TL) zebrafish embryos at 1.5 and 24 hpi. (a) Schematic showing the site of DSPC-LNP (*i.v.)* injection within embryonic zebrafish (2 dpf) and imaging timeframe. DSPC-LNPs contained DiD (Cy5, 0.1 mol%) as fluorescent lipid probe and unlabeled eGFP mRNA (capped) payload. *Injected dose*: ~10 mM lipid, ~0.2 mg/kg mRNA. *Injection volume*: ~1 nL. CCV – common cardinal vein. (b,c) Whole embryo (10x magnification) and tissue level (40x magnification) views of DSPC-LNP biodistribution and eGFP expression within the embryonic zebrafish at 1.5 hpi. DSPC-LNPs were mostly freely circulating, confined to and homogenously distributed throughout the vasculature of the embryo at 1.5 hpi. Low level embryo autofluorescence (GFP channel) within the yolk sac and pigment cells of the embryo is highlighted. (d,e) Whole embryo and tissue level views of DSPC-LNP biodistribution and eGFP expression within the embryonic zebrafish at 24 hpi. Widespread eGFP expression throughout the embryo indicates non-specific uptake of DSPC-LNPs in many different cell types. eGFP expression within macrophages (based on location and morphology) highlighted with white arrowheads. Scale bars: 200 µm (whole embryo) and 50 µm (tissue level).

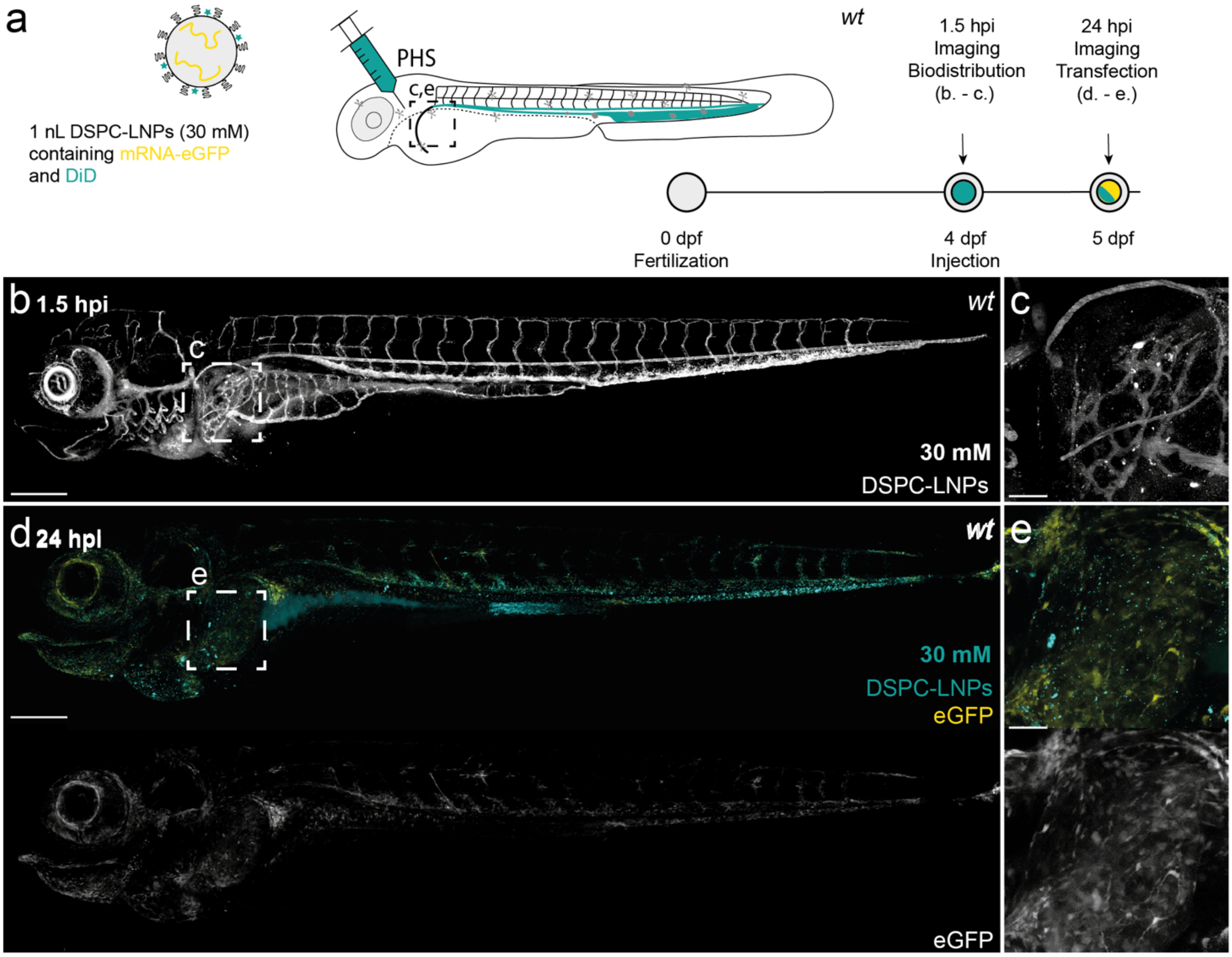

Figure S5. DSPC-LNP (30 mM) biodistribution and mRNA expression within wildtype (AB/TL) embryonic zebrafish. (a) Schematic showing the site of srLNP injection (*i.v.*) within embryonic zebrafish (4 dpf). DSPC-LNPs contained DiD (approx. 0.1 mol%) as fluorescent lipid probe and unlabeled, eGFP mRNA (capped) payload. Injection and imaging timeframe. *Injected dose*: ~30 mM lipid, ~0.6 mg/kg mRNA. *Injection volume*: 1 nL. PHS – primary head sinus. (b,c) Whole embryo (10x magnification) and tissue level (40x magnification, liver region) views of DSPC-LNP biodistribution (DiD, cyan) at 1.5 hpi. DSPC-LNPs were mainly freely circulating throughout the vasculature of the four-day old embryo at 1.5 hpi. Within the liver region, individual fluorescent punctae associated with DSPC-LNP accumulation are most likely due to macrophage uptake. (d,e) Whole embryo (10x magnification) and tissue level (40x magnification, liver region) views of DSPC-LNP biodistribution and eGFP expression within the embryonic zebrafish at 24 hpi. At this timepoint, DSPC-LNPs demonstrate widespread biodistribution and resultant eGFP expression throughout the embryo, including within the liver, but no preferential hepatic accumulation of LNPs nor enhanced, liver specific eGFP expression. Scale bars: 200 µm (whole embryo) and 50 µm (tissue level).

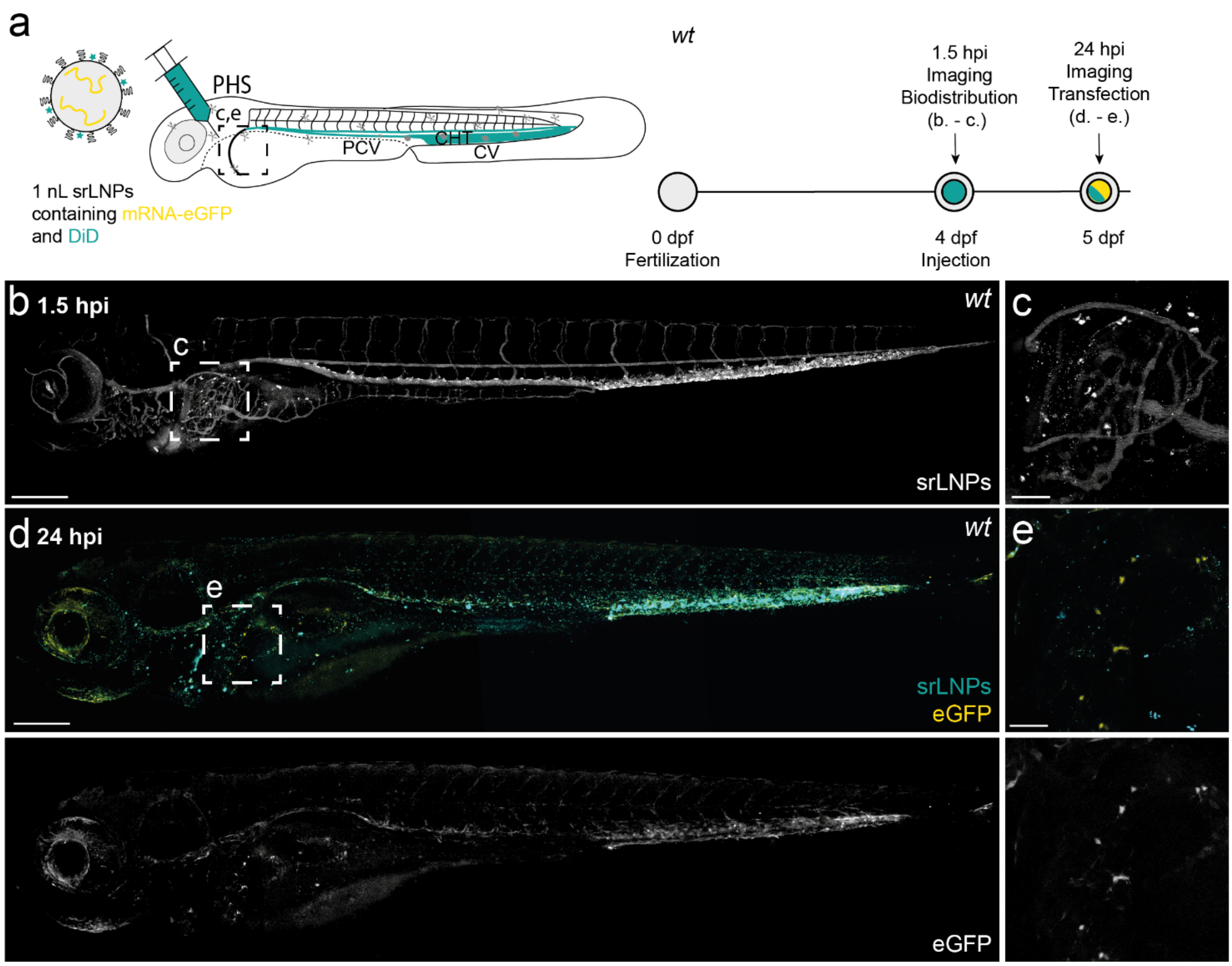

Figure S6. srLNP (30 mM) biodistribution and mRNA expression within wildtype (AB/TL) embryonic zebrafish. (a) Schematic showing the site of srLNP injection (*i.v.*) within embryonic zebrafish (4 dpf). srLNPs contained DiD (approx. 0.1 mol%) as fluorescent lipid probe and unlabeled, eGFP mRNA (capped) payload. Injection and imaging timeframe. *Injected dose*: ~30 mM lipid, ~0.6 mg/kg mRNA. *Injection volume*: 1 nL. PHS – primary head sinus. (b,c) Whole embryo (10x magnification) and tissue level (40x magnification, liver region) views of srLNP biodistribution (DiD, cyan) at 1.5 hpi. srLNPs were mainly associated with SECs within the PCV, CV and CHT of the four-day old embryo at 1.5 hpi. Due to the higher injected dosage, a significant fraction of srLNPs are also observed in circulation, possibly due to saturation of Stabilin receptors. Within the liver region, individual fluorescent punctae associated with srLNP accumulation are most likely due to macrophage uptake. (d,e) Whole embryo (10x magnification) and tissue level (40x magnification, liver region) views of srLNP biodistribution and eGFP expression within the embryonic zebrafish at 24 hpi. srLNPs remain predominantly localized within the PCV, CV and CHT at 24 hpi, with exogenous eGFP expression mainly restricted to this region of the five day-old embryo. Within the liver region, eGFP fluorescence is restricted to a handful of individual cells and does not evidently colocalize with srLNP-associated fluorescence (DiD). From these images, it is not clear whether eGFP fluorescence within the liver is due to macrophage uptake (possibly distal from the liver, and following macrophage migration), embryo autofluorescence or uptake within an alternative cell type. Scale bars: 200 µm (whole embryo) and 50 µm (tissue level).

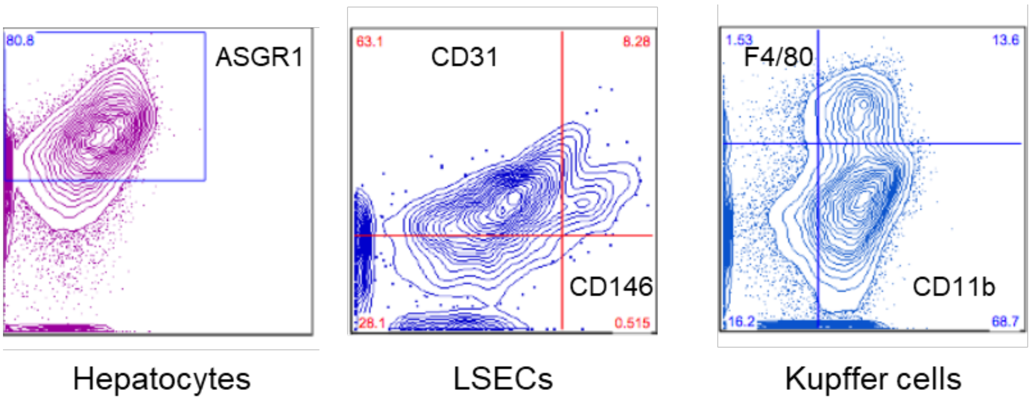

Figures S7. Detection of major cell types in the liver microenvironment. Representative flow cytometry density plots illustrate the detection of specific hepatic cell types following liver perfusion and cell harvesting.

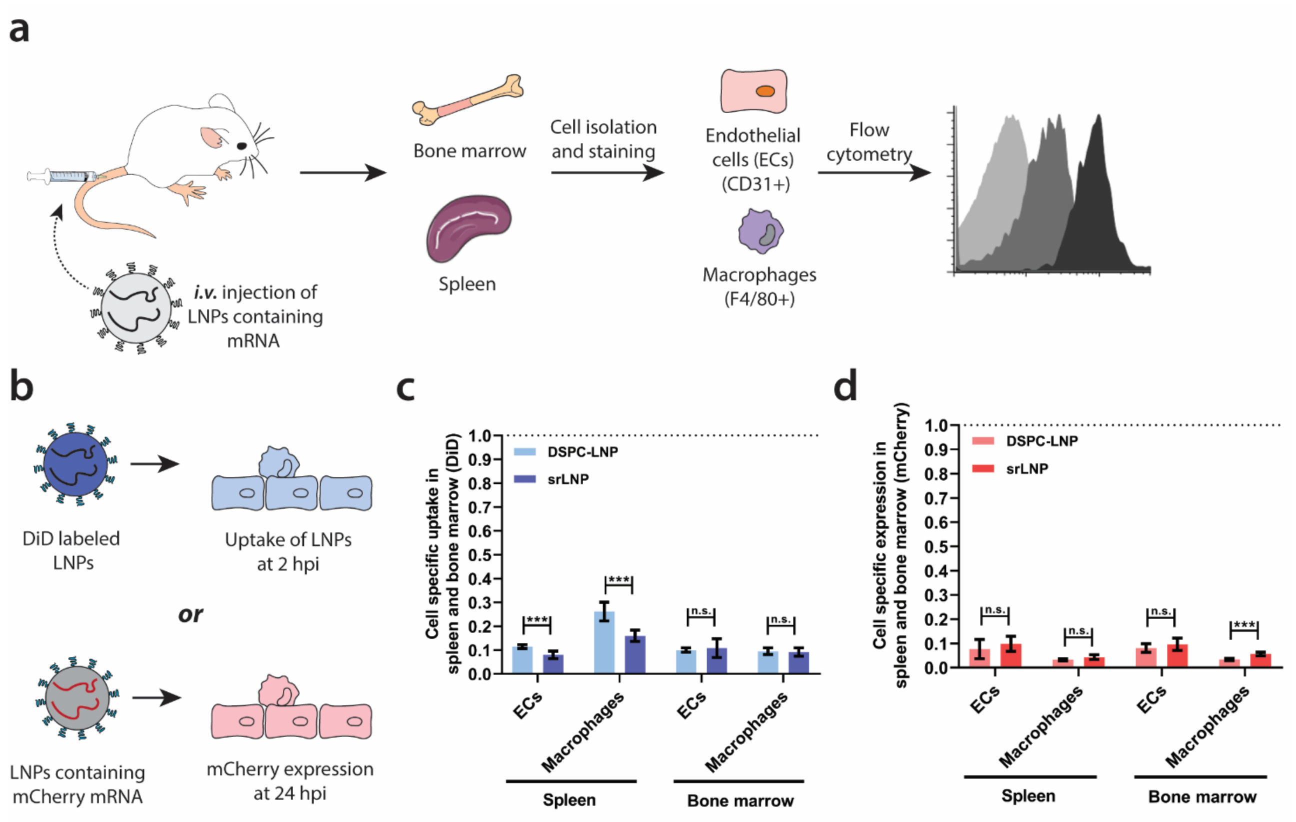

Figure S8. LNP uptake and functional mRNA delivery within different splenic cell types following i.v. administration in wildtype mice. (a) Schematic illustrating the procedure to isolate different splenic and bone marrow cell types to independently determine LNP targeting and functional mRNA delivery. Following intravenous LNP injection (*i.v.*), splenic and bone marrow cells were isolated and stained with specific antibodies (in parentheses), and flow cytometry used to analyze LNP uptake or gene expression in endothelial cells (ECs) and macrophages. (b) For biodistribution studies, LNPs contained DiD (0.5 mol%) as fluorescent probe. Cellular uptake of DSPC-LNP and srLNP was assessed 2 hpi. *Injected dose*: 42.75 mg/kg total lipid. For transfection studies, LNPs contained mCherry-mRNA. *Injected dose:* 0.25 mg/kg mRNA. In all cases, n = 6; representing 3 separate spleen or bone marrow tissue samples from 2 mice. (c) Cell specific uptake quantified by DiD fluorescence normalized to the uptake of DSPC-LNP in liver hepatocytes. (d) Cell specific expression of mCherry fluorescence normalized to the expression of DSPC-LNP in liver hepatocytes. Bars and error bars in **c** and **d** represent mean ± s.d. Statistical significance was evaluated using a two-tailed unpaired Student's t-test. n.s.= not significant $p > 0.01$, * $p < 0.01$ ** $p < 0.01$, *** $p < 0.001$. Exact P values for **c**: Splenic ECs P= 0.00652, splenic macrophages P= 0.000285, bone marrow ECs P= 0.622, bone marrow macrophages P= 0.719. Exact P values for **d**: Splenic ECs P= 0.308, splenic macrophages P= 0.0379, bone marrow ECs P= 0.251, bone marrow macrophages P= $6.29*10^{-5}$.

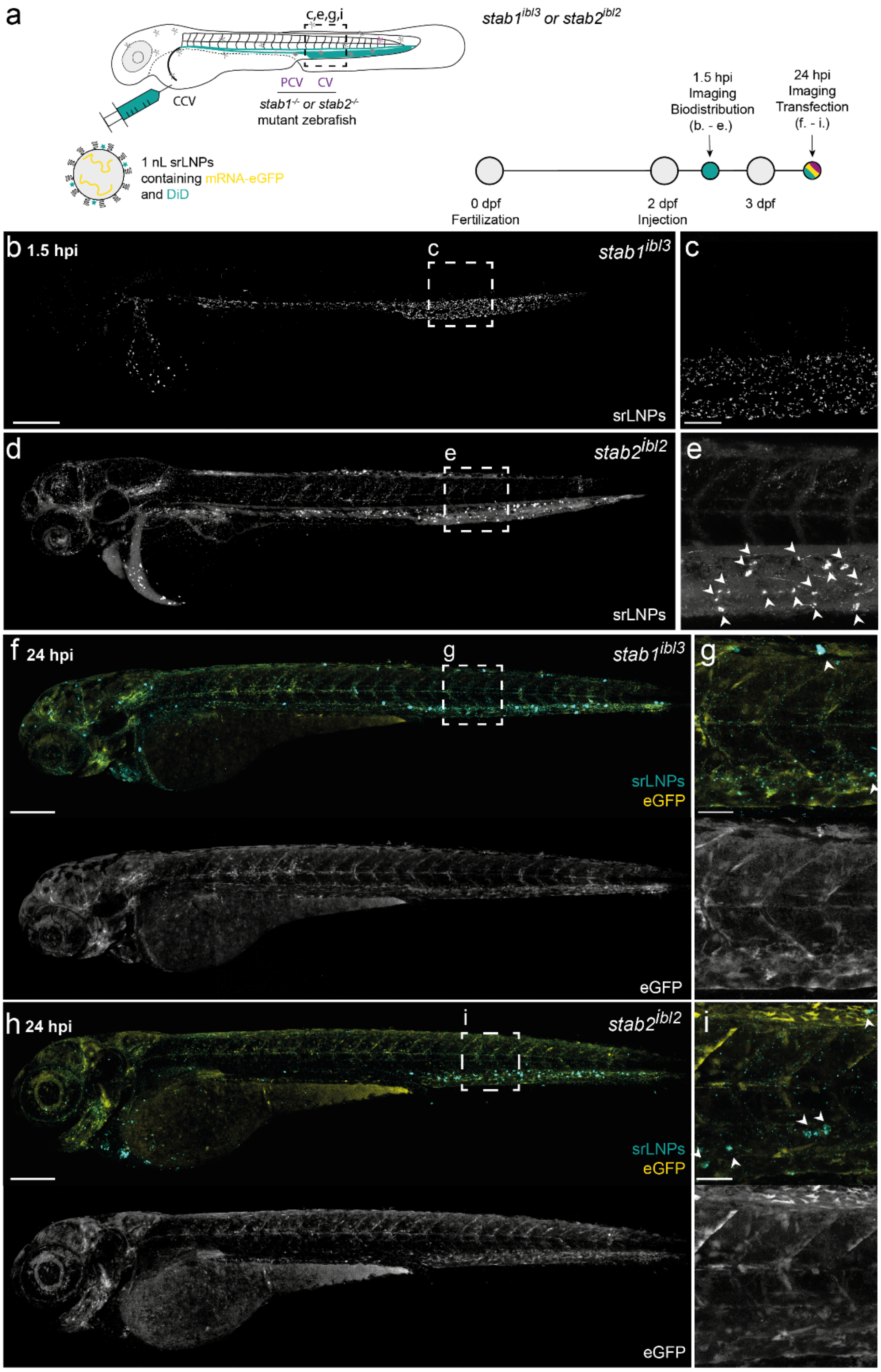

Figure S9. Biodistribution of srLNPs within single knock-out (*stab1*$^{-/-}$ *or stab2*$^{-/-}$) mutant embryos at 1.5 hpi. (a) Schematic showing the site of LNP injection (*i.v.*) within single knockout (*stab1*^{ibl3} or *stab2*^{ibl2}) [1,3] zebrafish embryos (2 dpf) and imaging timeframe. LNPs contained DiD (approx. 0.1 mol%) as fluorescent lipid probe and an unlabeled, eGFP mRNA (capped) payload. *Injected dose*: ~10 mM lipid, ~0.2 mg/kg mRNA. *Injection volume*: ~1 nL. CV – cardinal vein; PCV – posterior cardinal vein; CCV – common cardinal vein. (b,c) Whole embryo (10x magnification) and tissue level (40x magnification) views of srLNP biodistribution within a *stab1*$^{-/-}$ mutant embryo (2 dpf) at 1.5 hpi. In this KO, srLNPs accumulated within the CHT and CV of the zebrafish embryo, presumably *via* Stabilin-2-mediated recognition and uptake within SECs. (d,e) Whole embryo (10x magnification) and tissue level (40x magnification) views of srLNP biodistribution within a *stab2*$^{-/-}$ mutant embryo (2 dpf) at 1.5 hpi. In this KO, srLNPs were mostly freely circulating indicative of reduced interactions with SECs (now lacking *stab2*) and of the minor contribution of

Stabilin-1 in the recognition and uptake of srLNPs. Within the CHT, srLNPs still accumulate within blood resident macrophages (white arrowheads) confirming that srLNP uptake within macrophages is not exclusively reliant on Stabilin-2. (f,g) Whole embryo (10x magnification) and tissue level (40x magnification) views of eGFP expression within a embryonic *stab1*$^{-/-}$ mutant (3 dpf) at 24 hpi.(h-i) Whole embryo (10x magnification) and tissue level (40x magnification) views of eGFP expression within a embryonic *stab2*$^{-/-}$ mutant (3 dpf) at 24 hpi. Scale bars: 200 µm (whole embryo) and 50 µm (tissue level).

Supporting Table

Formulation	mRNA	Fluorescent lipid	% of Fluorescent Lipid	Avg. Size (nm)	PDI	Zeta Potential (mV)	EE (%)	n
DSPC-LNP	eGFP	DOPE-LR	0.2	82.5 ± 2.0	0.085 ± 0.027	-5.5 ± 1.2	95 ± 2	3
srLNP	eGFP	DOPE-LR	0.2	89.2 ± 5.7	0.094 ± 0.023	-21.9 ± 2.5	88 ± 3	3
DSPC-LNP	eGFP	DiD	0.1	82.5 ± 3.4	0.081 ± 0.026	-4.1 ± 1.6	95 ± 1	3
srLNP	eGFP	DiD	0.1	94.7 ± 4.0	0.102 ± 0.016	-17.5 ± 2.4	91 ± 2	3
DSPC-LNP	eGFP	-	-	82.5 ± 4.0	0.103 ± 0.037	-4.0 ± 0.9	93 ± 1	2
srLNP	eGFP	-	-	86.6 ± 6.7	0.108 ± 0.006	-19.0 ± 1.1	91 ± 4	2
DSPC-LNP	mCherry	DiD	0.1	79.9 ± 5.2	0.072 ± 0.038	-3.7 ± 1.8	94 ± 3	2
srLNP	mCherry	DiD	0.1	92.7 ± 3.5	0.102 ± 0.012	-18.8 ± 2.1	89 ± 4	2
DSPC-LNP	Cy5-mRNA	DOPE-LR	0.2	87.0 ± 3.5	0.090 ± 0.016	-3.9 ± 0.9	95 ± 3	2
srLNP	Cy5-mRNA	DOPE-LR	0.2	94.1 ± 2.3	0.096 ± 0.01	-16.2 ± 1.2	91 ± 2	2
DOPC	-	DOPE-LR	0.2	96.3 ± 4.0	0.095 ± 0.011	-6.1 ± 1.1	-	2
DOPC+ApoE-peptide	-	DOPE-LR	0.2	104.7 ± 3.8	0.075 ± 0.012	14.7 ± 1.6	-	2

Supporting Table S1. Composition, size (as measured by DLS), polydispersity (PDI), surface charge (as measured by zeta potential), RNA encapsulation efficiency (as measured by RiboGreen assay) and reproducibility of all LNP and liposome formulations used in this study.

Materials and Methods

Reagents

Dimethylformamide (DMF), piperidine, acetic anhydride, pyridine, trifluoroacetic acid (TFA) and acetonitrile (MeCN) were purchased from Biosolve (Valkenswaard, The Netherlands). N,N-diisopropylethylamine (DIPEA), and Oxyma were obtained from Carl Roth GmbH & Co (Karlsruhe, Germany). Dichloromethane (DCM) and diethyl ether were supplied by Honeywell (Amsterdam, The Netherlands). Fmoc-Rink Amide AM resin was obtained from IRIS Biotech GmbH (Marktredwitz, Germany). All amino acids were supplied by NovaBioChem, (Zwijndrecht, The Netherlands), a subsidiary of Merck. 1,2-distearoyl-*sn*-glycerol-3-phosphocholine (DSPC), 1,2-distearoyl-*sn*-glycero-3-phospho-(1'-rac-glycerol) (DSPG), 1,2-dioleyl-*sn*-glycerol-3-phosphocholine (DOPC) and 1,2-dimyristoyl-rac-glycero-3-methoxypolyethylene glycol-2000 (DMG-PEG2k) were purchased from Avanti Polar Lipids (Alabaster, USA) or Lipoid GmbH (Ludwigshafen, Germany). All other chemicals were purchased from Merck (Zwijndrecht, The Netherlands). (6Z,9Z,28Z,31Z)-heptatriaconta-6,9,28,31-tetraen-19-yl-4-(dimethylamino) butanoate (DLin-MC3-DMA) was synthesized as described.[4] 3-azido-5-cholestene (**1**) was synthesized as described.[5] CleanCap eGFP (5moU) mRNA, CleanCap mCherry (5moU) mRNA and CleanCap Cyanine 5 eGFP (5moU) mRNA were purchased from TriLink Biotechnologies (San Diego, USA) or Tebu-bio (Heerhugowaard, The Netherlands). Apolipoprotein E from human plasma (SRP6303) was obtained from Sigma-Aldrich.

Synthesis of N-succinyl-3-amino-5-cholestene (3)[6]

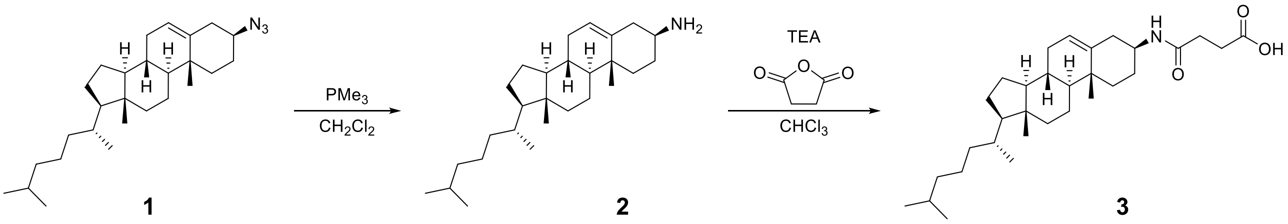

3-azido-5-cholestene (**1**, 1240 mg, 3 mmol) was dissolved in 30 mL dry DCM and the flask placed under a nitrogen atmosphere. A 1M solution of trimethylphosphine (12 mL, 12 mmol, 4 eq.) in toluene was added and the mixture was stirred for 21 hours. The reaction was quenched by the addition of 25 mL 1M sodium hydroxide solution, followed by vigorous stirring for 30 minutes. The mixture was transferred to a separating funnel, organic phase collected and the aqueous phase further extracted with 25 mL dichloromethane. The organic phases were combined and washed with 100 mL water (2x), 100 mL brine, and the organic phase dried over anhydrous magnesium sulfate. After concentration *in vacuo,* the resulting white powder was dried under high vacuum overnight to yield cholesteryl-amine (**2**) in quantitative yield.

Cholesteryl-amine **2** (440 mg, 1.14 mmol) was combined with succinic anhydride (342 mg, 3.4 mmol, 2.98 eq.) in 30 mL $CHCl_3$ and warmed to dissolve the mixture. Triethylamine (315 µL, 2,3 mmol, 2 eq.) was then added and the solution stirred for 16 hours. The reaction mixture was then washed with 50 mL 5% acetic acid solution in water (2x), 50 mL water (2x), 50 mL brine, and the organic phase dried over anhydrous magnesium sulfate. The mixture was concentrated *in vacuo* and filtered through a silica gel plug (eluent; 1:1 dichloromethane/ethyl acetate + 1% acetic acid). Concentration *in vacuo* yielded cholesteryl-4-amino-4-oxobutanoic acid (**3**) as a white powder (408 mg, 0.84 mmol, 74%).

^{1}H NMR (400 MHz, DMSO): δ 12.05 (s, 1H), 7.76 (d, *J* = 7.8 Hz, 1H), 5.34 – 5.20 (m, 1H), 3.45 – 3.38 (m, 1H), 2.39 (t, *J* = 6.9 Hz, 2H), 2.26 (t, *J* = 6.9 Hz, 2H), 2.09 (d, *J* = 8.2 Hz, 2H), 2.00 – 1.86 (m, 2H), 1.80 (d, *J* = 12.3 Hz, 2H), 1.65 – 1.27 (m, 13H), 1.16 – 0.78 (m, 24H), 0.65 (s, 3H).

Peptide synthesis

Solid-phase peptide synthesis was performed at a 0.1 mmol scale with Fmoc-Rink Amide AM resin (0.64 mmol/g) on a Liberty Blue™ Automated Microwave Peptide Synthesizer. Fmoc-deprotection was achieved using 20% piperidine in DMF, and coupling reactions using DIC as activator and Oxyma as base. The final Fmoc-group was deprotected and the resin was washed with DMF (3x) and DCM (3x) respectively and stored at 4 °C prior to further use.

Chol-NH-ApoE_peptide synthesis and purification

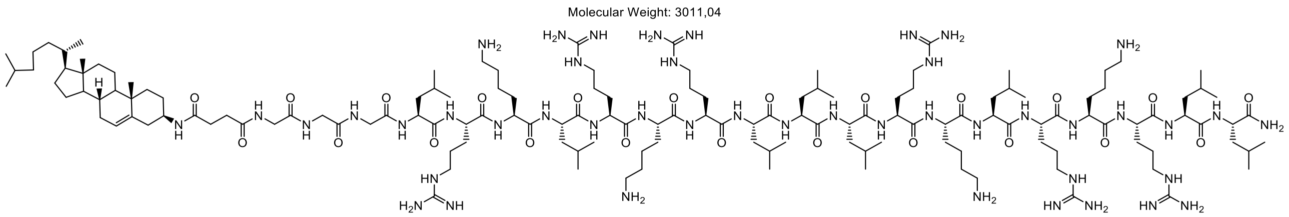

4
Chol-NH-GGG-LRKLRKRLLLRKLRKRLL
Chol-NH-apoE-peptide

To the synthesized peptide on solid support (0.025 mmol) in a fritted syringe was added cholesteryl-4-amino-4-oxobutanoic acid (**3**, 0.1 mmol, 4 eq.), HATU (0.1 mmol, 4 eq.), DIPEA (0.5 mmol, 20 eq.) and dimethylformamide (5 mL) and the mixture agitated overnight at room temperature. The liquids were filtered from the resin and the resin was washed with dimethylformamide (3 x 5 mL) and dichloromethane (3 x 5 mL). Cleavage from the resin was performed by the addition of a mixture of TFA : TIPS : water (95 : 2.5 : 2.5 vol%, 5 mL) and agitation at room temperature for 2 hours. The reaction was precipitated using ice-cold diethyl ether (45 mL) and the precipitate was collected by centrifugation (3000 *g*, 30 min), suspended in water and lyophilized. Purification was performed by reversed-phase HPLC on a Kinetic® Evo C18 column (5 µm, 100 Å, 150 mm x 21.2 mm) with a Shimadzu system comprising two LC-8A pumps and an SPD-10AVP UV-vis detector (operating at 220 nm). The cholesterol peptide-conjugate was purified using a gradient of 30–90% B, (where B is MeCN containing 0.1% TFA, and A is water with 0.1% TFA) over 20 minutes with a flow rate of 10 ml min^{-1}, eluting at 11.3-12.0 minutes. The collected fractions were analyzed using LC-MS, pooled

and freeze-dried. LC-MS was performed on a Kinetic® Biphenyl column (2.6 µm, 100 Å, 100 mm x 4.6 mm) using a Thermo Scientific™ Dionex™ Ultimate 3000 UHPLC (with a UV-VIS detector operating at 220 nm) coupled to a TSQ Quantum access MAX electron spray ionization (ESI) mass spectrometer operating with a ionization range of 140-2000 Da. Identification was performed using a gradient of 30–90% B, (where B is MeCN containing 0.1% TFA, and A is water with 0.1% TFA) over 13 minutes with a flow rate of 1 ml min^{-1}. Calculated mass: $[M+H]^{+}$ = 1506.0 Da ; $[M+2H]^{2+}$ = 1004.3 Da, Detected mass: $[M+H]^{+}$ = 1505.44 Da $[M+2H]^{2+}$ = 1003.1 Da.

Liposome Formulation

DOPC liposomes (with and without incorporated Chol-NH-ApoE-peptide, 5 mol%) were formulated in 20 mM HEPES buffer (pH = 7.3) at a total lipid concentration of 5 mM. DOPC and Chol-NH-ApoE-peptide, as stock solutions in chloroform (10 mM), were combined to the desired molar ratios and dried to a film, first under a stream of N_2 followed by the removal of trace solvents *in vacuo* for >1 h. Lipid films were hydrated and large unilamellar vesicles formed through extrusion at room temperature (Mini-extruder, Avanti Polar Lipids, Alabaster, US). Hydrated lipids were passed 11 times through a 100 nm polycarbonate (PC) membrane (Nucleopore Track-Etch membranes, Whatman). All liposome formulations were stored at 4°C and used within 2 days.

Lipid Nanoparticle (LNP) Formulation

Lipid nanoparticles entrapping mRNA were formulated as previously described.[7] In brief, lipid components (MC3, cholesterol, DSPC or DSPG, and PEG-lipid) were dissolved in ethanol. For biodistribution studies, the non-exchangeable tracer DiD or DOPE-LR was added to lipid mixtures at a concentration of 0.1 mol% or 0.2 mol% respectively. The mRNA was dissolved in 25 mM sodium acetate or sodium citrate buffer (pH 4). The two solutions were rapidly mixed (N/P ratio of 6) using a T-junction mixer (total flow rate of 20 mL/min, flow rate ratio of 3:1 v/v). The resulting LNP formulation was dialyzed overnight against PBS (pH 7.4), sterile filtered, and concentrated using 10K MWCO centrifugal filters (Amicon® Ultra, Merck). Entrapment efficiency and mRNA concentration were analyzed using the Quant-iT Ribogreen RNA assay (Life Technologies, Burlington, ON). Total

lipid concentrations were measured using the Cholesterol E Total-Cholesterol assay (Wako Diagnostics, Richmond, VA). mRNA doses within embryonic zebrafish were calculated using an estimated average weight of 1 mg per embryo, independent of developmental stage, and an injection volume of 1 nL.

LNP incubation with apolipoprotein E

LNPs were prepared as described above and normalized to a total lipid concentration of ~20 mM in PBS. Subsequently LNPs were mixed in a 1:1 volumetric ratio with solution of human apolipoprotein E (apoE) in PBS (5 mg/mL) and incubated at 37 °C for 1 hour. The mixture of LNPs (~10 mM) and apoE (2.5 mg/mL) were injected into embryonic zebrafish as described above.

LNP and liposome biophysical characterization

LNP and liposome sizes and zeta potentials were measured using a Malvern Zetasizer Nano ZS (software version 7.13, Malvern Panalytical). For DLS (operating wavelength = 633 nm), measurements were carried out at room temperature in 20 mM HEPES buffer (pH = 7.3) for liposomes, and in 1x PBS (pH = 7.4) for LNPs, at a total lipid concentration of approx. 100 µM. Zeta potentials were measured at 500 µM total lipid concentration, using a dip-cell electrode (ZEN1002, Malvern) for liposomes and in a folded capillary cell (DTS1070, Malvern) for LNPs, at room temperature. All reported DLS measurements and zeta potentials are the average of three measurements.

Cryo-electron Microscopy Imaging and Quantification

Vitrification of concentrated (~10 mM) LNPs was performed using a Leica EM GP operating at 21°C and 95% humidity. Sample suspensions were placed on glow discharged 100 µm lacey carbon films supported by 200 mesh copper grids (Electron Microscopy Sciences). Optimal results were achieved using a 60 second pre-blot and a 1 second blot time. After vitrification, sample grids were maintained below −170 ◦C and imaging was performed on a Tecnai T12 (ThermoFisher) with a biotwin lens and LaB6 filament operating at 120 keV equipped with an Eagle 4K x 4K CCD camera (ThermoFisher).

Images were acquired at a nominal underfocus of -2 to -3 μm (49,000× magnification) with an electron dose of ~2000 $e^{-}\cdot nm^{-2}$. Images were processed and particle size was quantified using the Fiji distribution of ImageJ.[8] For quantification, particle sizes were determined on particles present in amorphous vitrified water and obtained from a triplicate of assemblies (~150-200 particles per assembly per formulation). Generation of frequency distribution graphs was performed using GraphPad Prism (v 6.0).

Zebrafish Husbandry and Injections

Zebrafish (*Danio rerio*, strain AB/TL) were maintained and handled according to the guidelines from the Zebrafish Model Organism Database (http://zfin.org) and in compliance with the directives of the local animal welfare committee of Leiden University. Fertilization was performed by natural spawning at the beginning of the light period, and eggs were raised at 28.5 °C in egg water (60 μg/ mL Instant Ocean sea salts). In addition to wild-type (AB/TL) embryos, previously established *Tg(mpeg1:mCherry)*^{gl23} [9], *stab2*^{ibl2},[3] *stab1*^{ibl3} and *stab2*^{ibl2}*stab1*^{ibl3} [1] zebrafish lines were also used in this study. Fluorescently labelled LNPs or liposomes were injected into 54-96 hours post fertilization (hpf) zebrafish embryos following the detailed protocol described in reference [10]. Embryos were anesthetized in 0.01% tricaine and embedded in 0.4% agarose containing tricaine before injection. To improve reproducibility of microangiography experiments, 1 nL volume were calibrated and injected into the common cardinal vein (2-3 dpf) or primary head sinus (4 dpf). A small injection space was created by penetrating the skin with the injection needle and gently pulling the needle back, thereby creating a small pyramidal space in which the liposomes or LNPs were injected. Successfully injected embryos were identified through the backward translocation of venous erythrocytes and the absence of damage to the yolk ball. Selected zebrafish embryos successfully injected were kept in egg water at 28.5 degrees until later imaging (1.5 or 24 hours post injection, hpi).

Zebrafish confocal imaging acquisition and processing

Zebrafish embryos were randomly picked from a dish of 10-30 successfully injected embryos to be imaged after 1.5 or 24 hpi. Confocal z-stacks were captured on a Leica TCS SPE or SP8 confocal microscope, using a 10x air objective (HCX PL FLUOTAR), a 40x water-immersion objective (HCX APO L) or 63x water-immersion objective (HC PL APO CS). For whole-embryo views, 3 or 4 overlapping z-stacks were captured to cover the complete embryo. Laser intensity, gain and offset settings were identical between stacks and when comparing samples per experiment. Images were processed using the Fiji distribution of ImageJ.[8] Confocal image stacks (raw data) are available upon reasonable request.

Mouse husbandry, injection protocol and cell isolation

All mouse protocols were approved by the Canadian Animal Care Committee and conducted in accordance with relevant guidelines and regulations. Mice were maintained on a regular 12-hour light/12- hour dark cycle in a specific pathogen-free animal facility at UBC. For wildtype, C57Bl6 male mice aged between 8 to 10 weeks were used throughout. Stabilin-2 knockout mice (*stab2*$^{-/-}$ KO) were developed at the University of Tokyo, as previously described,[11] and provided by the Harris lab (University of Nebraska, USA).

These mice were divided into groups of 2 and either received intravenous (*i.v*) injection of LNP-mRNAs (either DSPC-LNPs or srLNPs) or PBS as a negative control. For biodistribution studies, LNPs entrapping luciferase mRNA were labelled with 0.5 mol% DiD as fluorescent lipid marker. Injections were performed at 42.75 mg/kg total lipid and mice were sacrificed at 2 hpi. For gene expression studies, LNPs encapsulating mRNA coding for the fluorescent reporter gene mCherry were used, injections were performed at 0.25 mg/kg mRNA dose, and mice were sacrificed at 24 hpi. Mice were anesthetized using a high dose of isofluorane followed by CO_2. Trans-cardiac perfusion was performed as follows: once the animals were unresponsive, a 5 cm medial incision was made through the abdominal wall, exposing the liver and heart. While the heart was still beating, a butterfly needle connected to a 30 mL syringe loaded with pre-warmed Hank's Balanced Salt Solution (HBSS,

Gibco) was inserted into the left ventricle. Next, the liver was perfused with perfusion medium (HBSS, supplemented with 0.5 mM EDTA, Glucose 10mM and HEPES 10mM) at a rate of 3 mL/min for 10 min. Once liver swelling was observed, a cut was performed on the right atrium and perfusion was switched to digestion medium (DMEM, Gibco supplemented with 10% fetal bovine serum (FBS, Gibco) and 1% penicillin streptomycin (Gibco) and 0.8 mg/mL Collagenase Type IV, Worthington) at 3 mL/min for another 10 min. At the end of the perfusion of the entire system, as determined by organ blanching, the whole liver and spleen were dissected and transferred to 50 mL Falcon tubes containing 10 mL ice cold (4°C) perfusion media and placed on ice. Next, isolation of hepatic cell types (*i.e.* hepatocytes, Kupffer cells (KCs) and liver sinusoid endothelial cells (LSECs)) was performed following density gradient-based separation. Briefly, the liver was transferred to a Petri dish containing digestion medium, minced under sterile conditions, and incubated for 20 min at 37℃ with occasional shaking of the plate. Cell suspensions were then filtered through a 40 µm mesh cell strainer to eliminate any undigested tissue remnants. Primary hepatocytes were separated from other liver residing cells (LRCs) by low-speed centrifugation at 500 rpm with no brake. The supernatant containing mainly LRCs was pelleted using low speed (3000 rpm) centrifugation at 4°C, aliquoted and washed twice with ice cold PBS containing 2% FBS. The pellet containing mainly hepatocytes was collected, washed at low speed and placed on ice. Phenotypic detection using monoclonal antibodies, assessment of LNP delivery and mRNA expression on cells liver cells was performed immediately after isolation to avoid changes in gene regulation, polarization and dedifferentiation.[12] LNP biodistribution across individual RES cell types of the spleen (*i.e.* endothelial cells and macrophages) were also characterized. Here, the spleen was also dissected and placed into a 40 µm mesh cell and mashed through the cell strainer into the petri dish using the plunger end of the syringe. The suspended cells were transferred to a 15 mL Falcon tube and centrifuged at 1000 rpm for 5 minutes. The pellet was resuspended in 1 mL ACK lysis buffer (Invitrogen) to lyse the red blood cells, aliquoted in FACS buffer and stained with antibodies as described below to identify splenic endothelial cells and macrophages.

FACS analysis

Cell aliquots were resuspended in 300 µL FACS staining buffer (FBS 2%, sodium azide 0.1% and ethylenediaminetetraacetic acid (EDTA 1mM)) followed by staining with fluorescence tagged antibodies. Prior to staining, cells were first labeled with anti-mouse CD16/CD32 (mouse Fc blocker, Clone 2.4G2) (AntibodyLab, Vancouver, Canada) to reduce background. Hepatocytes were identified following staining with primary mouse antibody detecting ASGR1 (8D7, Novus Biologicals) followed by goat polyclonal secondary antibody to mouse IgG2a labeled to PE-Cy7 (BioLegend). Kupffer cells were identified with CD11b –FITC or PE (Invitrogen) and F4/80high labeled to APC. LSECs were identified with CD146-VioBlue (Miltenyi Biotec) and CD31-PE-Cy7 (Abcam). Spleen macrophages and endothelial cells were detected using appropriate antibodies and identified as CD11bhigh and CD31+ve cells following antibody labeling as described. The data were acquired using a LSRII flow cytometer and the FACSDiva software and analyzed by FlowJo following acquisition of at least 10,000 events after gating on viable cell populations. LNP-mRNA delivery or transfection efficacy were assessed based on the relative mean fluorescence intensity of DiD or mCherry positive cells, respectively, measured on histograms obtained from gated cell populations.

Statistics and Reproducibility

Frequency distributions for LNP size, derived from cryo-EM micrographs, and mean ± standard deviation was obtained using Prism (v8.1.1, GraphPad Software, Inc.). All zebrafish experiments were repeated at least twice, with the exception of Supporting Figure 3 (performed once). All replicate experiments were performed using freshly prepared LNPs or liposomes. All replicate experiments were successful and confirmed the presented data. For all experiments performed in embryonic zebrafish, at least four embryos were randomly selected (from a pool of 10-30 successfully injected embryos) and analyzed (low resolution microscopy). All selected embryos showed consistent results and confirmed the presented data. From these embryos, at least one embryo was selected for high resolution, confocal microscopy. No statistical analysis was performed on acquired zebrafish data. Statistical analysis for mouse studies was performed by a Student's t-test with a correction for

multiple comparisons using the Holm-Sidak method using Prism (v8.1.1, GraphPad Software, Inc.). All data represent at $n \geq 2$ independent measurements. Comparisons were considered significant at $P < 0.01$.

Data Availability

Data supporting the findings of this paper (*e.g.* raw confocal Z-stacks) are available from the corresponding authors upon reasonable request. Source data for all mice experiments (Figs 6, S9 and S10) can be provided as a single Excel file upon request.

Supporting Data

LC-MS spectrum of Chol-NH-ApoE_peptide

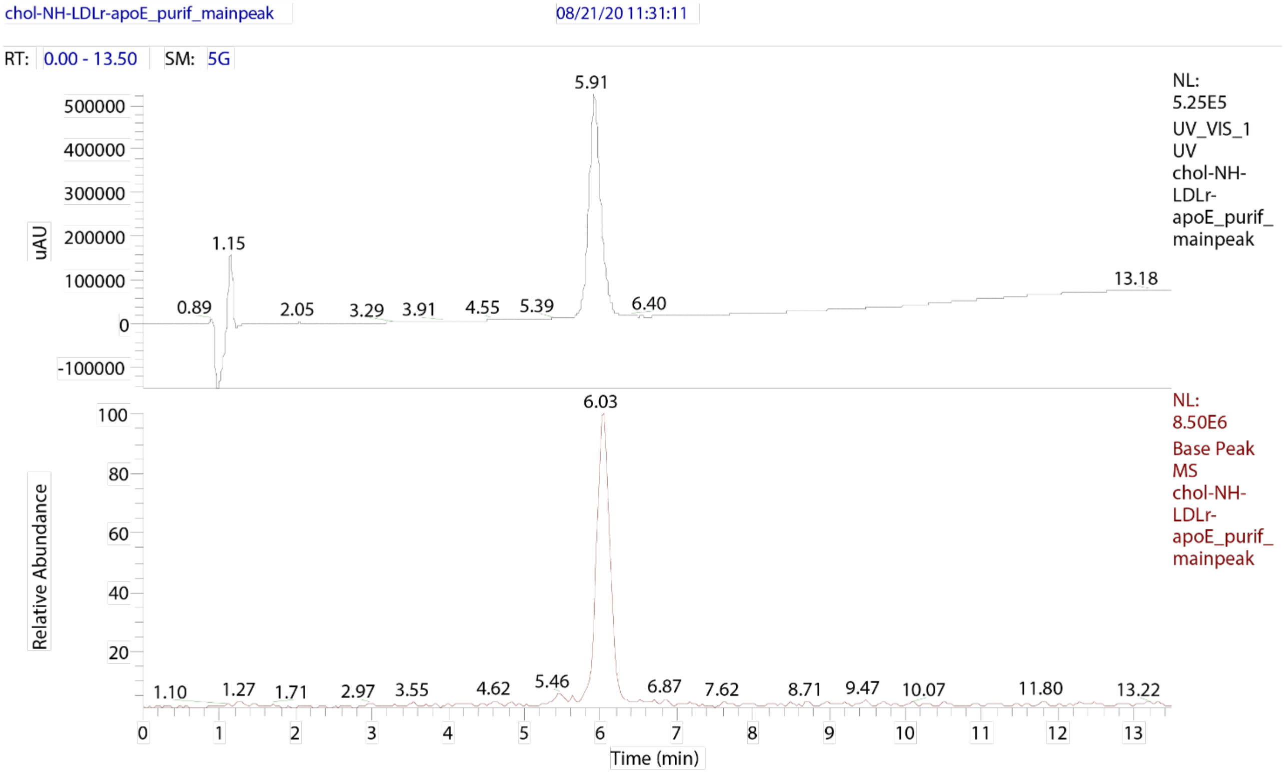

chol-NH-LDLr-apoE_purif_mainpeak
Type: Unknown ID: RA1 Row: 1
Sample Name:
Study:
Client:
Laboratory:
Company:

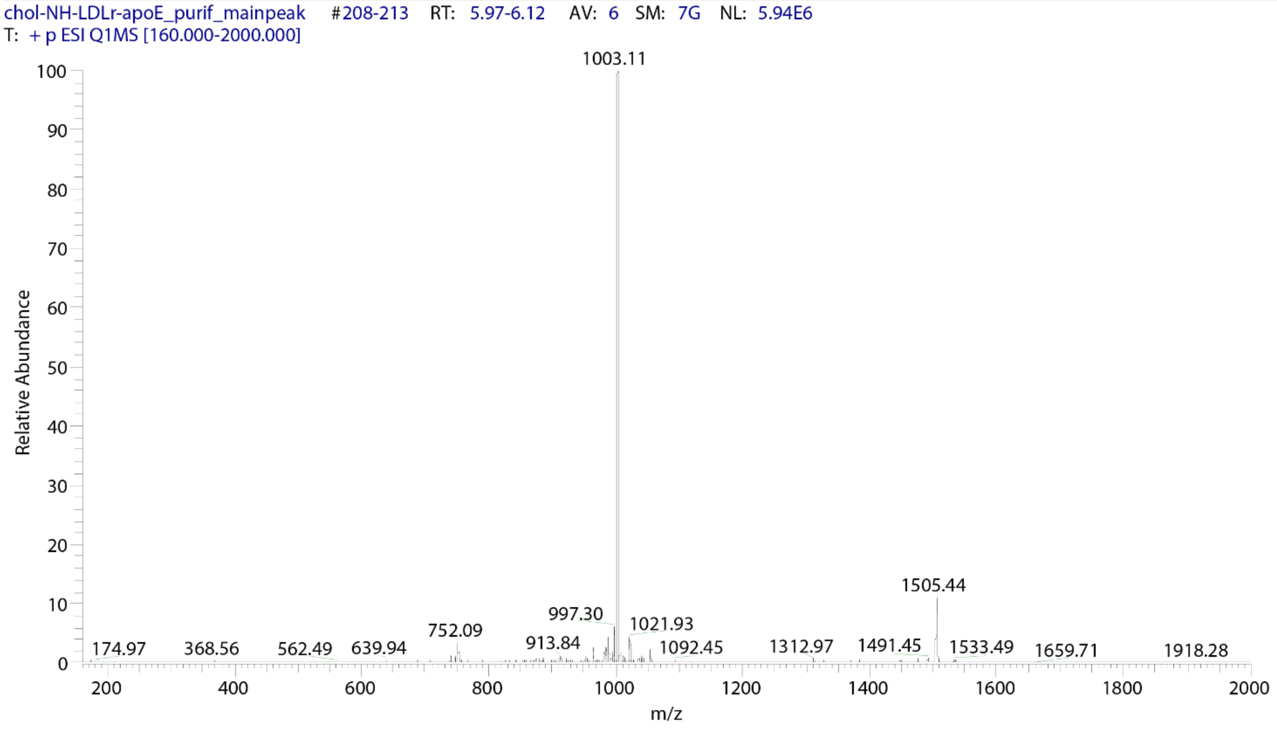

References

[1] G. Arias-Alpizar, B. Koch, N. M. Hamelmann, M. A. Neustrup, J. M. J. Paulusse, W. Jiskoot, A. Kros, J. Bussmann, *Nanomedicine Nanotechnology, Biol. Med.* **2021**, *34*, 102395.

[2] C. M. Miller, A. J. Donner, E. E. Blank, A. W. Egger, B. M. Kellar, M. E. Østergaard, P. P. Seth, E. N. Harris, *Nucleic Acids Res.* **2016**, *44*, 2782.

[3] F. Campbell, F. L. Bos, S. Sieber, G. Arias-Alpizar, B. E. Koch, J. Huwyler, A. Kros, J. Bussmann, *ACS Nano* **2018**, *12*, 2138.

[4] M. Jayaraman, S. M. Ansell, B. L. Mui, Y. K. Tam, J. Chen, X. Du, D. Butler, L. Eltepu, S. Matsuda, J. K. Narayanannair, K. G. Rajeev, I. M. Hafez, A. Akinc, M. A. Maier, M. A. Tracy, P. R. Cullis, T. D. Madden, M. Manoharan, M. J. Hope, *Angew. Chemie - Int. Ed.* **2012**, *51*, 8529.

[5] Q. Sun, S. Cai, B. R. Peterson, *Org. Lett.* **2009**, *11*, 567.

[6] G. A. Daudey, H. R. Zope, J. Voskuhl, A. Kros, A. L. Boyle, *Langmuir* **2017**, *33*, 12443.

[7] J. A. Kulkarni, D. Witzigmann, J. Leung, R. van der Meel, J. Zaifman, M. M. Darjuan, H. M. Grisch-Chan, B. Thöny, Y. Y. C. Tam, P. R. Cullis, *Nanoscale* **2019**, *11*, 9023.

[8] J. Schindelin, I. Arganda-Carreras, E. Frise, V. Kaynig, M. Longair, T. Pietzsch, S. Preibisch, C. Rueden, S. Saalfeld, B. Schmid, J.-Y. Tinevez, D. J. White, V. Hartenstein, K. Eliceiri, P. Tomancak, A. Cardona, *Nat. Methods* **2012**, *9*, 676.

[9] F. Ellett, L. Pase, J. W. Hayman, A. Andrianopoulos, G. J. Lieschke, *Blood* **2011**, *117*, e49.

[10] G. Arias-Alpizar, J. Bussmann, F. Campbell, *Bio-protocol* **2021**, *11*, e4173.

[11] Y. Hirose, E. Saijou, Y. Sugano, F. Takeshita, S. Nishimura, H. Nonaka, Y.-R. Chen, K. Sekine, T. Kido, T. Nakamura, S. Kato, T. Kanke, K. Nakamura, R. Nagai, T. Ochiya, A. Miyajima, *Proc. Natl. Acad. Sci.* **2012**, *109*, 4263 LP.

[12] M. Severgnini, J. Sherman, A. Sehgal, N. K. Jayaprakash, J. Aubin, G. Wang, L. Zhang, C. G. Peng, K. Yucius, J. Butler, K. Fitzgerald, *Cytotechnology* **2012**, *64*, 187.
